# Allosteric coupling asymmetry mediates paradoxical activation of BRAF

**DOI:** 10.1101/2023.04.18.536450

**Published:** 2023-04-19

**Authors:** Damien M. Rasmussen, Manny M. Semonis, Joseph M. Muretta, Andrew R. Thompson, David D. Thomas, William C.K. Pomerantz, Nicholas M. Levinson

**Affiliations:** 1 Department of Pharmacology, University of Minnesota, Minneapolis, MN, 55455; 2 Department of Biochemistry, Molecular Biology, and Biophysics, University of Minnesota, Minneapolis, MN,55455; 3 Department of Chemistry, University of Minnesota, Minneapolis, MN, 55455

## Abstract

Both first-generation αC-out and newer αC-in RAF inhibitors paradoxically activate BRAF kinase at subsaturating concentrations. Paradoxical activation by αC-in inhibitors is linked to the formation of BRAF dimers, but why activation rather than inhibition occurs remains unclear. We used biophysical methods tracking BRAF conformation and dimerization combined with thermodynamic modeling to define the allosteric coupling mechanism underlying paradoxical activation. Allosteric coupling between αC-in inhibitors and BRAF dimerization is both extremely strong and highly asymmetric, with the first inhibitor contributing the bulk of dimer promotion. This asymmetric allosteric coupling mechanism results in the induction of dimers in which only one protomer is inhibited while the other is activated. The type II class of RAF inhibitors currently in clinical trials are more asymmetrically coupled and possess greater activation potential than older type I inhibitors. ^19^F NMR data demonstrate that the BRAF dimer displays dynamic conformational asymmetry, with only a subset of protomers locked in the αC-in state, explaining how the conformational effects of drug binding can efficiently drive BRAF dimerization and activation at substoichiometric concentrations.

The protein kinase BRAF is a central component of the MAPK (RAF-MEK-ERK) signaling pathway and plays a critical role in the regulation of eukaryotic cell growth, proliferation, and survival^1,2^. In quiescent cells, BRAF exists in an autoinhibited monomeric state within the cytosol^3–5^. Upon growth factor signaling, BRAF monomers are recruited to the plasma membrane by RAS-GTP where they are allosterically activated by dimerization. BRAF dimerization triggers a conformational change of the regulatory αC-helix from an inactive αC-out state to an active αC-in state^6^. Activated BRAF dimers initiate a phosphorylation cascade in which MEK and ERK are sequentially activated by successive phosphorylation events^1^.

Mutations in BRAF play a major role in human cancers, most notably in melanoma where approximately half of all cases are driven by BRAF^7^. The most prevalent BRAF mutation is V600E, which accounts for more than half of BRAF mutations across all cancer types and 95% of cases in melanoma^8^. The V600E mutation confers constitutive kinase activity by disrupting autoinhibitory interactions within the cytoplasmic pool of BRAF, allowing it to phosphorylate its substrate MEK independently of upstream signaling^9,10^. The FDA-approved BRAF inhibitors vemurafenib, dabrafenib, and encorafenib show remarkable initial responses in V600E-driven metastatic melanoma patients, but clinical resistance emerges rapidly^11–13^. These drugs potently inhibit V600E BRAF monomers, but paradoxically activate BRAF dimers, leading to increased MAPK signaling^14–16^. Consequently, mechanisms that promote the formation of BRAF dimers, including receptor tyrosine kinase (RTK) activation, RAS mutations, and BRAF splice variants, cause clinical resistance to these inhibitors^17–19^. Similar mechanisms driving dimer formation explain why BRAF inhibitors have limited single-agent efficacy in BRAF-driven colorectal cancer^20,21^. Paradoxical activation of BRAF is also responsible for the formation of secondary cutaneous carcinomas in many patients^22^.

The FDA-approved inhibitors fail to block the BRAF dimer because they recognize the inactive αC-out state of the kinase and are unable to bind to the αC-in state adopted by dimeric BRAF^23^. This discovery prompted the development of a new class of inhibitors that recognize the active αC-in state^24,25^. Remarkably, despite binding to both protomers of the BRAF dimer in x-ray structures^24^, these αC-in inhibitors can still induce paradoxical activation in cell lines^14,26–29^. This ability has been linked to the induction of BRAF dimers^30^, but the molecular mechanisms underlying this activation remain elusive. Answering this question could shed light on the failure of several of these drugs in clinical trials^31,32^ and inspire development of new inhibitors that fully circumvent paradoxical activation.

Here we use biophysical techniques tracking BRAF conformational change, dimerization, and activation to provide a comprehensive molecular mechanism for paradoxical activation of BRAF by αC-in inhibitors. BRAF dimer induction by αC-in inhibitors occurs through an asymmetric allosteric coupling mechanism in which the first inhibitor binding event drives BRAF dimerization strongly, with only small additional contributions from the second inhibitor molecule. This asymmetry drives kinase activation by promoting the formation of partially-occupied BRAF dimers in which only a single protomer is bound to inhibitor. We also show that the BRAF dimer is dynamically asymmetric, with the αC-helix locked in the αC-in state in only a subset of molecules and in conformational exchange in the remaining ones. This observation provides an explanation for how inhibitor-driven induction of the αC-in state in only one protomer can effectively promote BRAF dimerization.

## RESULTS

### αC-in inhibitors drive BRAF dimerization through asymmetric allosteric coupling

**Figure 1:**
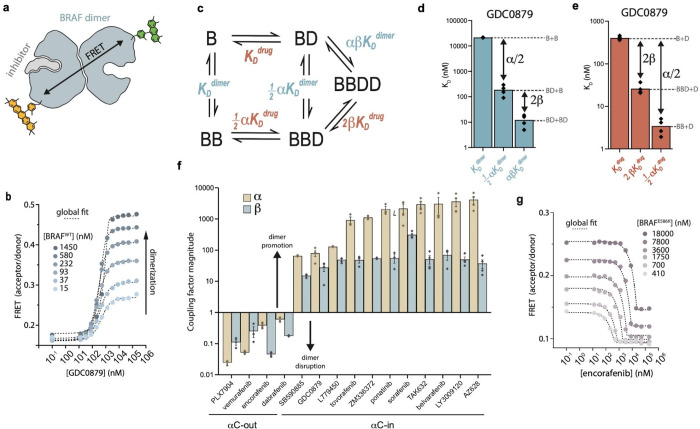
αC-in inhibitors drive BRAF dimerization through asymmetric allosteric coupling. **a,** Schematic of the intermolecular FRET sensor used to quantify BRAF dimerization. **b,** Representative intermolecular FRET experiments measuring BRAF dimerization by αC-in inhibitor at different BRAF concentrations. Dashed lines represent the global fit to the model shown in panel c. Data for all αC-in inhibitors are shown in Supplementary Figure 2. **c,** Model describing inhibitor-induced BRAF dimerization used to globally fit the FRET data. B represents apo BRAF monomer, BD drug/inhibitor-bound monomer, BB apo dimer, BBD dimeric BRAF with one bound inhibitor molecule, and BBDD dimeric BRAF with two inhibitor molecules bound. These biochemical species are linked by the equilibrium dissociation constants described in the main text. **d,e,** Equilibrium dissociation constants for dimerization (panel d) and inhibitor binding (panel e) determined from global fitting analysis of GDC0879 FRET experiments shown in panel b. Allosteric coupling factors α and β describe the coupling of BRAF dimerization to the first and second inhibitor binding events, respectively (see Methods). Data for all inhibitors are shown in Supplementary Figure 3. **f,** Allosteric coupling factors α and β are shown for all RAF inhibitors. Error bars represent the mean ± s.e.m.; n≥3 independent experiments, each performed in duplicate. **g,** Representative intermolecular FRET experiments measuring disruption of BRAFE586K dimerization by αC-out inhibitor at increasing BRAF concentrations. Dashed lines represent the global fit to the thermodynamic model shown in panel c.

We used intermolecular FRET to quantify inhibitor-induced BRAF dimerization. A previously-validated construct of the BRAF kinase domain bearing 16 solubilizing mutations^5,33^ (hereafter referred to as “BRAF”) was labeled on K547C with donor (AF488) or acceptor (AF568) fluorophores, mixed at equal molar ratios, and dispensed into multiwell plates (Fig. 1a). Dimerization was inferred from increases in the acceptor/donor (A/D) ratio (Fig. 1b). The dimerization affinity of BRAF $\left({K_{D}}^{\text{dimer}}\right)$ was found to be 21.8±1.7 μM, in agreement with previously reported values^30^ (Supplementary Fig. 1a,b). We then examined BRAF dimerization in the presence of a diverse set of 11 αC-in (3 type I, 8 type II) and 4 αC-out RAF inhibitors (Supplementary Table 1). All αC-in inhibitors induced large increases in the A/D ratio, consistent with inhibitor-induced BRAF dimerization (Fig. 1b and Supplementary Fig. 2). The change in A/D ratio with BRAF concentration observed at saturating inhibitor indicated dimerization in the nanomolar range, representing at least two orders of magnitude enhancement over baseline dimerization.

We globally fit the FRET data to a thermodynamic model describing inhibitor-induced BRAF dimerization^34^ (Fig. 1c). In this model, dimerization is described by three equilibrium dissociation constants quantifying apo BRAF dimerization, dimerization with one inhibitor bound, and dimerization with two inhibitors bound. In turn, inhibitor binding is described by three dissociation constants quantifying binding to monomeric BRAF, binding to apo dimeric BRAF, and binding to dimeric BRAF with one inhibitor already bound (Fig. 1c and Methods). The FRET data were mapped onto the model using one fluorescence coefficient to describe the low-FRET monomeric forms of BRAF and a second coefficient to describe the high-FRET dimeric forms of BRAF (see Methods). With the dimerization affinity of apo BRAF (${K_{D}}^{\text{dimer}}$) constrained to a value of 21.8 μM (see above), global fitting produced well-constrained values for all other dissociation constants (Fig. 1d and Supplementary Fig. 1b,c and Supplementary Fig. 3).

The incorporation of both BRAF and drug dose responses, combined with the constraints imposed by a physically realistic model (see Methods), allow the global fit analysis to provide greater insight into inhibitor-driven dimerization than simpler analyses. For instance, by explicitly separating inhibitor-driven dimerization into two steps, the contributions of the first and second inhibitor molecules can be resolved and are quantified by the allosteric coupling factors α and β, respectively. The factor α can be thought of as the degree to which baseline dimerization (${K_{D}}^{\text{dimer}}$) is enhanced by a single bound inhibitor, while the factor β quantifies any additional stabilization from a second inhibitor molecule. (Fig. 1d).

For all 11 αC-in inhibitors, global fitting revealed that the enhancement of BRAF dimerization is both remarkably strong and highly asymmetric, with the large majority of dimer promotion provided by the first inhibitor binding event (α factors as high as $\left.10^{3}-10^{4}\right)$, and the contributions from the second inhibitor molecule far smaller $\left(\beta \right.$ factors of $10^{1}-10^{2}$, Fig. 1f and Supplementary Fig. 3c). Additionally, the type II inhibitors, including the clinical drugs ponatinib, belvarafenib, and tovorafenib, are stronger and more asymmetric dimerizers than the type I inhibitors. These patterns of allosteric coupling also mean that the αC-in inhibitors bind poorly to BRAF monomers, moderately well to partially occupied dimers, and most tightly to apo BRAF dimers (Fig. 1e and Supplementary Fig. 4). Indeed, the model predicts that most αC-in inhibitors bind monomers with $K_{D}$ values > 100 nM, outside the potency regime typically required for ATP-competitive kinase inhibitors. These predictions were confirmed in control intramolecular FRET experiments where drug binding was measured using a monomeric BRAF construct (BRAF^DB^) containing mutations that prevent dimerization (see Methods, supplementary fig. 5).

To verify that dimer promotion is primarily driven by α, we performed additional FRET experiments where the effects of β were eliminated by exploiting the active site mutation A481F known to block inhibitor binding^35^ (Supplementary Fig. 6a). In BRAF^A481F^ control experiments, the mutation indeed prevented inhibitor-induced dimerization (Supplementary Fig. 6b). BRAF^A481F^ labeled with acceptor and BRAF labeled with donor were mixed at equal molar ratios. Under these conditions, only the formation of BRAF^A481F^: BRAF heterodimers, which are capable of binding only one inhibitor molecule (biochemical species “BBD” in the model) and are thus driven only by α, should lead to observable FRET changes (Supplementary Fig. 6a). In these experiments, we observed inhibitor-driven increases in the A/D ratios similar in magnitude to those observed in a matched BRAF:BRAF experiment, indicating the formation of inhibitor-induced BBD dimers (Supplementary Fig. 7a). Fitting the BRAF concentration response at saturating inhibitor revealed dimerization affinities in the low nanomolar range for the type II inhibitors, representing an approximately 1000-fold increase over baseline dimerization affinity (Supplementary Fig. 7b), in good agreement with the α factors measured for type II inhibitors. The type I inhibitor GDC0879, which possesses a more modest α factor (Fig. 1f), failed to induce heterodimers in this experiment. These results are consistent with α being the dominant factor in inhibitor-induced BRAF dimerization.

We also tested the effects of four αC-out inhibitors on BRAF dimerization. All four disrupted BRAF dimerization to such an extent that no dimerization signal was observed at saturating inhibitor, preventing global fitting from converging to a constrained solution. To circumvent this, we used the oncogenic dimer interface mutation E586K (BRAF^E586K^) to enhance dimerization^10^, allowing us to obtain α and β factors for each αC-out inhibitor (Fig. 1f,g and Supplementary Fig. 8). This analysis showed that dimer disruption by the αC-out inhibitors is weaker than dimer promotion by the αC-in inhibitors, with total decreases in dimerization affinity of 1–2 orders of magnitude (Supplementary Fig. 4). The weakening is not distributed equally between α and β, with the former dominating for vemurafenib and PLX 7904 and the latter for dabrafenib and encorafenib (Fig. 1f). These results suggest a general tendency for both dimer-enhancing and dimer-breaking RAF inhibitors to be coupled to dimerization in an asymmetric manner.

### Allosteric asymmetry drives accumulation of partially occupied BRAF dimers that are catalytically active

**Figure 2.**
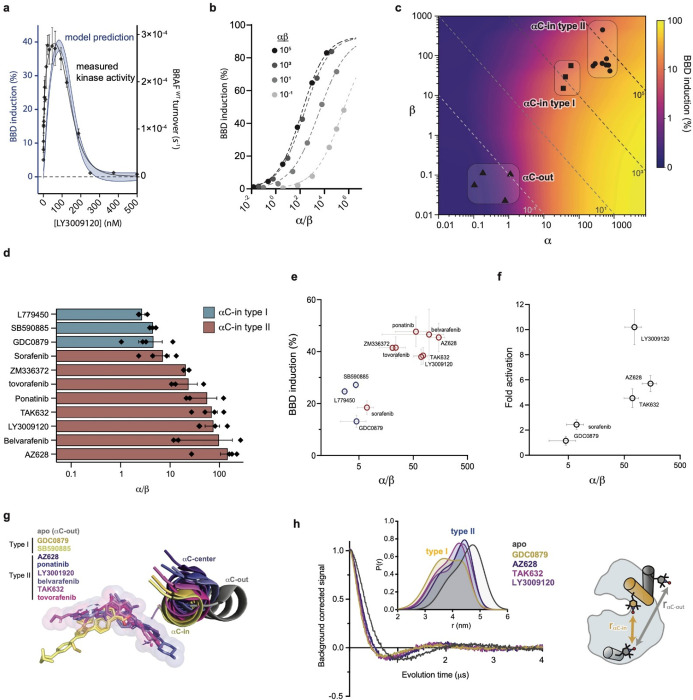
Allosteric asymmetry is the driving force for paradoxical activation by αC-in inhibitors. **a,** Representative BRAF kinase activity data (black diamonds, right y-axis) and simulations predicting the formation of partially occupied BBD dimers (blue line, left y-axis), induced by LY3009120. Activity data were fit to a bell-shaped dose-response curve (black line) and represent mean values ± s.e.m.; n=3 independent experiments each performed in duplicate. Activity data for other inhibitors are shown in Supplementary Figure 7. BBD induction curves were simulated using the thermodynamic model parameterized via global fitting of FRET data, and were fit to a bell-shaped dose-response curve. The thickness of the band represents the 95% Cl of the best-fit model from n=3 independent experiments. Simulations for other inhibitors are shown in Supplementary Figure 8. **b,** Simulations predicting BBD induction as a function of the α/β ratio at different overall magnitudes of dimerization enhancement (αβ). Simulations were performed using an artificially parametrized thermodynamic model where ${K_{D}}^{\text{dimer}}$ and ${K_{D}}^{\text{drug}}$ were kept constant and α and β were systematically varied. Data were fit to a variable slope (four parameter) dose-response curve. **c**, Landscape showing BBD induction over a range of α and β factors based on simulations from the artificially parametrized thermodynamic model. Inhibitors were mapped onto the landscape based on their experimentally determined α and β factors. Dashed lines represent curves shown in panel b. **d**, Allosteric coupling ratios (α/β) for αC-in type I (blue) and αC-in type II (red) inhibitors. Ratios were calculated from the global fitting analysis of FRET data (see Methods). Data represent the mean ± s.e.m.; n≥3 independent experiments each performed in duplicate. **e,** Simulated BBD induction magnitudes versus allosteric coupling ratio (α/β) for αC-in type I (blue) and αC-in type II (red) inhibitors. Data represent the mean \pm s.e.m.; n≥3 independent experiments each performed in duplicate. **f**, Inhibitor-induced fold increase in kinase activity as a function of the allosteric coupling ratio (α/β). Fold activation was calculated from the maximum activity at the peak relative to the DMSO control. Kinase activity data represent the mean ± s.e.m.; n=3 independent experiments each performed in duplicate. **g**, X-ray structures of BRAF bound to type I and type II inhibitors (PDB IDs: 2FB8, 4MNF, 5C9C, 6P3D, 6V34, 4RZW, 4KSP, 6XFP). Structures were aligned on the C-terminal lobe. **h,** DEER waveforms and Gaussian distance distributions (inset) for apo BRAF, type I inhibitor GDC0879 and the type II inhibitors AZ628, TAK632, and LY3009120. A schematic representing the labeling strategy used to track the movement of the αC-helix in DEER experiments is shown on the right.

To investigate the functional consequences of asymmetric inhibitor-induced dimerization, we used the experimentally parametrized thermodynamic models for each inhibitor to simulate the abundance of different BRAF species as a function of inhibitor concentration (see Methods). For all αC-in inhibitors, simulations predict a bell-shaped curve for the induction of partially occupied BBD dimers that increases with inhibitor concentration and peaks at approximately a 1:2 molar ratio of inhibitor to BRAF, before decreasing at higher concentrations due to the formation of fully saturated dimers (“BBDD” in the model) (Fig. 2a and Supplementary Fig. 9). We refer to the peak BBD concentration as the “BBD induction magnitude”.

To determine how the coupling factors α and β influence BBD dimer induction, the model was systematically adjusted to sample a broad range of α/β ratios while keeping apo BRAF dimerization affinity $\left({K_{D}}^{\text{dimer}}\right)$ and the total magnitude of dimerization enhancement (αβ) constant. This analysis showed that the BBD induction magnitude is primarily controlled by the ratio between α and β, or the degree of asymmetry in the allosteric model, with larger α/β ratios leading to greater induction (Fig. 2b). A separate analysis showed that the αβ product has little effect in the strong dimer enhancement regime represented by the αC-in inhibitors (αβ values > 10^3^), but that substantially weaker dimer inducers would require greater asymmetry to achieve the same BBD induction magnitude (Fig. 2b). We next simulated BBD formation over a wide range of α and β parameter space, allowing effects arising from the α/β ratio and αβ product to be compared simultaneously on a BBD induction landscape (Fig. 2c). Mapping the experimentally determined α and β factors for each inhibitor onto the BBD induction landscape revealed that BRAF inhibitors cluster into three unique regions (Fig. 2c). Notably, the type I and type II αC-in inhibitors are resolved into separate groups. With the exception of sorafenib, the larger α/β ratios of type II inhibitors translate into BBD induction magnitudes of 38–48% of total BRAF (Fig. 2d and Fig. 2e). In contrast, type I inhibitors have more modest α/β ratios, which translate into BBD induction magnitudes of only 13–27% (Fig. 2d and Fig. 2e). Together, these results indicate that type II inhibitors induce dimerization in a more asymmetric manner than type I inhibitors, resulting in a greater degree of BBD induction.

To confirm that the induction of BBD dimers leads to an increase in kinase activity, we used a fluorescence-based kinase activity assay to directly measure phosphorylation of MEK by BRAF. In this assay, type II inhibitors induced dose-dependent increases in BRAF activity up to 8-fold above the no-inhibitor control that agreed strikingly well with the simulated BBD induction curves (Fig. 2a and Supplementary Fig. 10). In contrast, the type I inhibitor GDC0879 induced only a marginal increase in BRAF activity (Fig. 2f), and the αC-out inhibitors vemurafenib and dabrafenib failed to induce any increase (Supplementary Fig. 10). These results support the model that paradoxical activation by αC-in inhibitors occurs due to asymmetric allosteric coupling that drives the accumulation of catalytically active BBD dimers. The degree of asymmetry dictates the magnitude of BBD dimer induction, with type II inhibitors exhibiting greater asymmetry and stronger activation than type I inhibitors.

The differential effects of type I and type II inhibitors could be caused by the induction of distinct kinase conformations. Although both type I and II inhibitors are typically thought to promote the αC-in state, the type II inhibitor ponatinib has been reported to instead stabilize an “αC-center” conformation that is intermediate between the αC-in and the αC-out states^28^. Analysis of x-ray structures of BRAF in complex with a set of type I and type II inhibitors suggested that these inhibitor classes indeed promote distinct αC-helix conformations, with the type I structures adopting the αC-in state and the type II structures spanning a range of conformations between αC-in and αC-out (Fig. 2g). To test whether these conformations are also sampled in solution, we performed double electron-electron resonance (DEER) spectroscopy on BRAF bound to type I or type II inhibitors, using one spin probe incorporated on the αC-helix (Q493C) and one on the αG-helix (Q664C). This labeling strategy yields shorter spin-spin distances for the αC-in state and longer distances for the αC out state (Fig. 2h, Supplementary Fig. 13). Distance distributions derived from fitting of the DEER data (see Methods) confirmed that the type II inhibitors AZ628, LY3009120, and TAK632 induce longer average spin-spin distances than the type I inhibitor GDC0879 (Fig. 2h). These observations point to a general tendency for type II inhibitors to stabilize intermediate αC-helix states, while type I inhibitors stabilize the canonical αC-in state. These different αC-helix conformations are coupled to changes in the N-lobe to C-lobe orientation, which is known to modulate BRAF dimerization^30^, likely contributing to the differential effects of type I and type II inhibitors on dimerization.

### The V600E mutation minimally perturbs BRAF conformation and allosteric coupling

**Figure 3.**
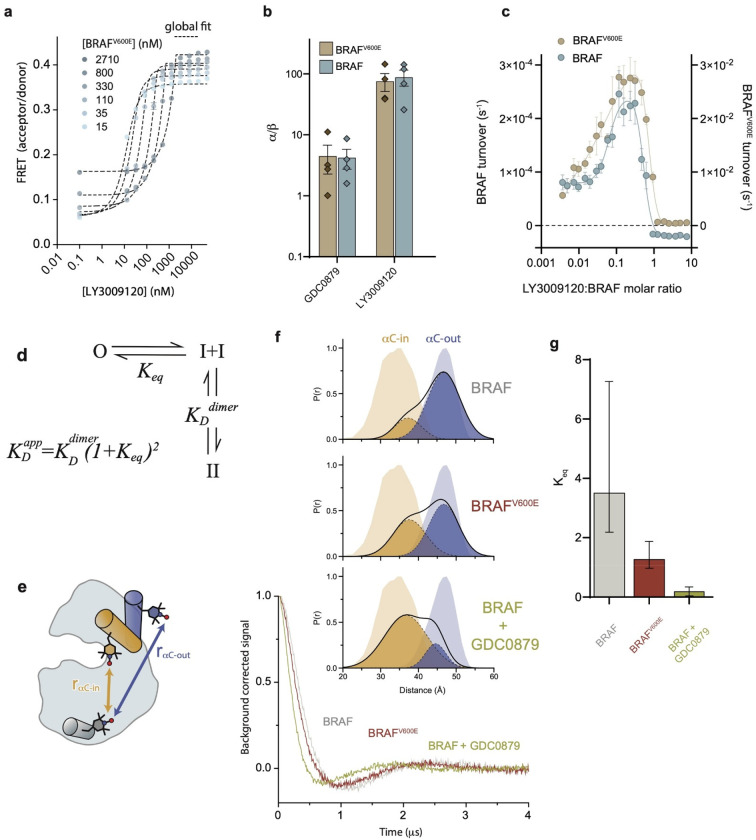
The V600E mutation minimally perturbs BRAF conformation and leaves the asymmetric allosteric coupling mechanism intact. **a,** Representative intermolecular FRET experiments measuring BRAF^V600E^ dimerization as a function of LY3009120 concentration. Dashed lines are the global fit to the thermodynamic model shown in Figure 1c. Data for additional inhibitors are shown in Supplementary Figure 9. **b,** Allosteric coupling ratios (α/β) for BRAF^V600E^ (brown) and BRAF (blue) with GDC0879 and LY3009120. Ratios were calculated from the global fitting analysis of FRET data (see Methods). Data represent the mean ± s.e.m.; n=4 independent experiments. Data for additional inhibitors are shown in Supplementary Figure 10b. **c,** Kinase activity of BRAF^V600E^ (brown, right y-axis) and BRAF (blue, left y-axis) as a function of LY3009120. Data represent the mean ± s.e.m.; n=3 independent experiments performed in duplicate. Data for additional inhibitors are shown in Supplementary Figure 10c. **d,** Allosteric model describing the coupling between the BRAF conformational equilibrium ($K_{\text{eq}}$) and the BRAF monomer-dimer equilibrium (${K_{D}}^{\text{dimer}}$). In this model the apparent BRAF dimerization affinity $\left({K_{D}}^{\text{app}}\right)$ is dependent on the conformational equilibrium between the αC-out state (O) and the αC-in state (I). **e,** A schematic representing the labeling strategy used to track the movement of the αC-helix in DEER experiments. The αG-helix (gray), inactive αC-out (blue), and active αC-in (yellow) states of the αC-helix are shown labeled with a nitroxide spin probe. The $r_{\alpha c\text{-in}}$ and $r_{\alpha c\text{-out}}$ labels represent the spin-spin distances of the active and inactive states, respectively. **f,** DEER experiments tracking the position of the αC-helix. Background-corrected DEER data (bottom panel) are shown for BRAF (gray), BRAF^V600E^ (red) and BRAF bound to GDC0879 (green). Two-Gaussian fits of the DEER data (top panels) are shown as solid black lines. Individual Gaussian components corresponding to the αC-in (yellow) and αC-out (blue) conformational states are overlaid on spin-spin distance simulations (transparent yellow and blue) calculated using MTSSLwizard (see Methods and Supplementary Figure 13). **g,** The BRAF conformational equilibrium ($K_{\text{eq}}=[\alpha C-out]/[\alpha C-in]$) represented in panel d was derived from the mole fractions calculated from the Gaussian fits of DEER data. Error bars represent the 75% Cls calculated from 5,000 simulations of Gaussian fits to the primary data.

Oncogenic BRAF mutations like V600E are thought to destabilize the αC-out state, thereby promoting the dimerization competent αC-in state^36^. We therefore reasoned that the V600E mutation might alter the allosteric coupling between inhibitor binding and BRAF dimerization. FRET dimerization experiments performed with the V600E mutant of BRAF (BRAF^V600E^), revealed a dimerization affinity of 3.1±0.3 μM, approximately 7-fold higher than BRAF (Fig. 3a and Supplementary Fig. 11). Global fitting revealed that the mutation does not substantially impact the remaining parameters of the allosteric coupling model. In particular, dimerization affinities of BRAF^V600E^ bound to one or two inhibitor molecules are similar to the respective values observed with BRAF, despite the stronger baseline dimerization affinity (Supplementary Fig. 11b). This is accounted for by an equivalent fold change in both α and β coupling factors of approximately 3.5-fold for type II inhibitors and 10-fold for the type I inhibitor GDC0879 (Supplementary Fig. 12a). Because the fold changes are approximately equal, the α/β ratio remains nearly identical to what is seen with BRAF (Fig. 3b and Supplementary Fig. 12b).

Consistent with these results, simulations with the experimentally-parametrized BRAF^V600E^ model predict bell-shaped BBD induction curves similar to those observed with BRAF, and kinase assays confirmed similar inhibitor-induced activity trends (Fig. 3c and Supplementary Fig. 12c) despite the higher basal kinase activity of the mutant. These results indicate that inhibitor-induced BRAF dimerization is largely unaffected by the V600E mutation, with BRAF^V600E^ exhibiting a similar asymmetric allosteric coupling mechanism that leads to paradoxical activation by αC-in inhibitors.

The limited effect of the V600E mutation can be rationalized by an allosteric model in which the apparent dimerization affinity of BRAF depends upon the equilibrium between the αC-in state, which can dimerize, and the αC-out state, which cannot (Fig. 3d). Based on this model, we reasoned that the modest enhancement of dimerization observed with BRAF^V600E^ arises due to a correspondingly subtle shift of the equilibrium towards the αC-in state. To test this model, we used DEER spectroscopy to probe the effects of the V600E mutation on the αC-in/αC-out equilibrium (Fig. 3e). DEER data collected with monomeric BRAF were best fit by a two-Gaussian distance distribution, corresponding to two spin-spin distances centered at 37.3 Å and 43.2 Å, similar to the simulated distances for the αC-in and αC-out states of 34.2 Å and 46.5 Å (Fig. 3f and Supplementary Fig. 13). The mole fractions of the αC-in and αC-out states revealed that in BRAF the conformational equilibrium favors the αC-out state with $K_{eq}=[\alpha C-out]/[\alpha C-in\;]=3.52$ (75% Cl:2.18, 7.26), consistent with the weak observed dimerization affinity (Fig. 3f,g). BRAF^V600E^ yielded spin-spin distances nearly identical to BRAF but with a greater sampling of the αC-in state, corresponding to $K_{eq}=1.29\;$(75%Cl:0.96, 1.95). The allosteric model predicts that this 2.7-fold change in $K_{\text{eq}}$ resulting from the V600E mutation should lead to a 3.9-fold change in ${{\text{K}}_{\text{D}}}^{\text{dimer}}$, in reasonable agreement with the 7-fold increase observed experimentally.

The addition of GDC0879 to BRAF resulted in a much greater shift towards the αC-in state, yielding a $K_{eq}=0.22$ (75% CI: 0.04,0.34) (Fig. 3g). These data confirm that αC-in inhibitors trigger a larger conformational shift than the V600E mutation, explaining at least in part their stronger enhancement of BRAF dimerization. Because of their larger conformational effects, inhibitor binding will also negate further shifts from the V600E mutation, explaining why the mutation has little effect on the allosteric coupling with inhibitors.

### The αC-helix in the BRAF dimer dynamically samples multiple conformational states

**Figure 4.**
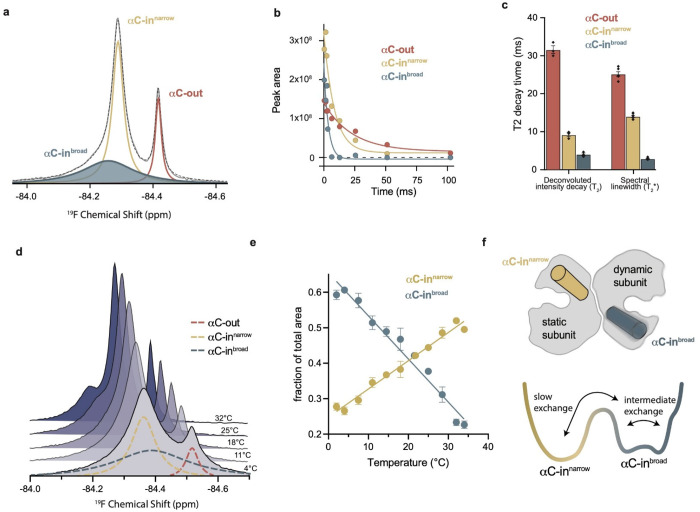
The αC-helix in the BRAF dimer dynamically samples multiple conformational states. **a,** ^19^F NMR spectrum of apo BRAF labeled on Q493C on the α C-helix with BTFA. The deconvoluted spectrum consists of three unique resonances that correspond to one αC-out state (red) and two αC-in states (blue and yellow). Resonance assignments are shown in Supplementary Figures 14 and 14 c. **b,** Representative ^19^F NMR T_2_ relaxation profiles showing the peak areas of individual deconvoluted peaks shown in panel a as a function of $T_{2}$ decay time. $T_{2}$ decay parameters for each component were extracted by single-exponential fits. **C,** Comparison of $T_{2}$ vs. $T_{2}*.T_{2}$ decay times calculated for each component as described in panel b are compared to ${\text{T}}_{2}*$ decay times calculated from the deconvoluted spectral linewidths of each component with the relationship ${\text{T}}_{2}*=1/(\text{π}\times \;\text{linewidth}\;)$. Data represent the mean ± s.e.m.; n=4 independent experiments. d, Variable temperature ^19^F NMR experiments. Spectra from lowest (light blue) to highest (dark blue) temperatures are overlaid with spectral deconvolutions described in panel a and are shown as dotted lines. **e,** Deconvoluted peak areas of αC-in^narrow^ (yellow) and αC-in^broad^ (blue) peaks from variable temperature ^19^F NMR experiments shown in panel **d.** Data represent the mean ± s.e.m.; n=3 independent experiments. **f,** A schematic model of dynamic heterogeneity in the BRAF dimer showing the relatively static αC-in^narrow^ (yellow) and more dynamic αC-in^broad^ (blue) states observed by ^19^F NMR experiments. This model can be further visualized with a free-energy diagram showing the αC-in^narrow^ and αC-in^broad^ states being almost equally populated at 20°C and in slow exchange with each other. Furthermore, the data are consistent with the αC-in^broad^ state consisting of multiple conformational states with relatively small energy barriers between them, thus giving rise to exchange broadening on the intermediate time scale.

To gain further insight into the mechanism underlying asymmetric allosteric coupling, we used ^19^F NMR to study the conformational dynamics of the BRAF αC-helix by incorporating the cysteine-reactive trifluoromethyl NMR probe 3-Bromo-1,1,1-trifluoroacetone (BTFA) at the same site used for DEER. Spectra of labeled apo BRAF showed two well-resolved resonances at −84.29 ppm and −84.42 ppm (Fig. 4a). Resonance assignment was achieved by adding saturating concentrations of ATP and AZ628, known to induce αC-out and αC-in states, respectively. From this, the upfield resonance was defined as the αC-out (monomeric) state and the downfield resonance as the αC-in (dimeric) state (Supplementary Fig. 14a). Increasing the concentration of BRAF led to an increase in the αC-in peak area and a decrease in the αC-out peak area that fit to a monomer-dimer equilibrium with a $K_{D}$ of 26.3 ±5.7 μM, in agreement with the FRET experiments (Supplementary Fig. 14b).

Spectral deconvolution of the apo BRAF data identified the existence of an additional broad resonance underneath the αC-in peak (Fig. 4a). The second αC-in resonance, referred to as “αC-in ^broad^”, is severely exchange broadened, with a fitted line width approximately five times that of the sharper overlapping αC-in resonance, hereafter referred to as “αC-in^narrow^” (Supplementary Fig. 14d). The presence of the αC-in ^broad^ state was independently detected in transverse relaxation ($T_{2}$) experiments, where the peak intensity of the αC-in resonance was measured as a function of the transverse magnetization evolution time and fit to an exponential decay model (Supplementary Fig. 14e). A double exponential fit was necessary to adequately describe these data (p<0.0001), demonstrating the presence of two overlapping species with distinct relaxation times. These experimental relaxation times were in good agreement with calculated relaxation times (T_2_*) derived from the deconvoluted line widths (Fig. 4b,c). The short relaxation time of the broad resonance is consistent with significant chemical exchange.

Four independent observations indicate that the αC-in^broad^ resonance arises from a dimeric species: 1) the chemical shift of this species is similar to that of the αC-in^narrow^ resonance (Fig. 4a), 2) spectral deconvolution revealed a concentration dependence for the area of the αC-in^broad^ peak that fits to a monomer-dimer equilibrium model closely agreeing with independent measurements (Supplementary Fig. 14b), 3) the αC-in^broad^ resonance is not observed in BRAF samples with dimer-disrupting interface mutations (Supplementary Fig. 14c), 4) $D_{2}O$ exchange experiments revealed equivalently large isotope shifts for the αC-in^narrow^ and αC-in^broad^ resonances (0.06 versus 0.08ppm), indicating a similar degree of solvent exposure of the probe, compared to a relatively small shift for the αC-out monomer peak (0.02 ppm) (Supplementary Fig. 14f).

Experiments performed at temperatures ranging from 2°C to 34°C revealed that, as the temperature is raised, the αC-in^broad^ peak width decreases, consistent with faster exchange kinetics (Fig. 4d,e). At the same time, the αC-in^broad^ peak area decreases and the αC-in^narrow^ peak area increases in a reciprocal manner, indicating a shifting equilibrium between the two dimeric states (Fig. 4d,e). The equilibrium constant for this process, derived from the ratio of the integrated areas of the αC-in ^broad^ and αC-in^narrow^ resonances, exhibits a roughly linear dependence on temperature (Fig 4e).

We propose the following interpretation of these results. Dimeric BRAF transitions between two states with strikingly different dynamics. In one state, the kinase is locked in the αC-in state, with the αC-helix relatively static (αC-in^narrow^). In the other more dynamic state, the αC-helix undergoes transitions between conformational substates on the μs-ms timescale (αC-in^broad^) (Fig. 4f). In contrast, transitions between the conformationally locked and dynamic states occur on the slow exchange timescale. Although our results do not define how the two states are distributed across the subunits of the dimer, the dynamic αC-in state is populated under all experimental conditions sampled. This indicates that BRAF dimers are not rigidly constrained in a symmetrical αC-in/αC-in state but are stable with only a subpopulation of protomers locked in the αC-in state. This explains how inhibitor binding to, and promotion of the αC-in state in, only a single protomer can drive dimerization of BRAF so efficiently, as observed in our FRET experiments. Thus, the dynamic heterogeneity of the BRAF dimer revealed by NMR may represent the underlying basis for the asymmetric interaction with αC-in inhibitors that drives paradoxical activation.

## DISCUSSION

The clinical experience with vemurafenib and dabrafenib in melanoma underscores the significance of paradoxical activation of BRAF, as it represents the mechanism responsible for secondary skin tumors in patients treated with these inhibitors^22^. Activation of BRAF by these αC-out inhibitors is attributed to the induction of negative allostery between BRAF protomers^37^ and the inability to occupy both protomers. This model fails to explain why αC-in inhibitors explicitly designed to avoid negative allostery and to bind both BRAF protomers still cause paradoxical activation. Here we use a series of spectroscopic approaches paired with thermodynamic modeling to describe the allosteric coupling between BRAF dimerization and inhibitor binding in molecular detail, yielding the first quantitative description of how αC-in inhibitors trigger paradoxical activation.

Our data reveal that inhibitor binding and dimerization are allosterically coupled to a remarkable degree, with αC-in inhibitors increasing BRAF dimerization affinity by many orders of magnitude. This allosteric coupling is not equally distributed across both protomers or inhibitor molecules, but is highly asymmetric, with the binding of the first inhibitor dominating the process of dimer induction. We demonstrate that this allosteric coupling asymmetry leads to inhibitor-driven increases in BRAF kinase activity through the formation of catalytically active BRAF dimers that are bound to only a single inhibitor. The degree of this asymmetry determines an inhibitor’s activating potential, with greater asymmetry leading to greater activation. In particular, type II αC-in inhibitors are coupled to BRAF dimerization in a more asymmetric manner and induce BRAF dimerization to a greater extent than type I inhibitors. We attribute these differences to the promotion of distinct αC-helix conformations and degrees of active site opening, which are known to be coupled to BRAF dimerization^30^. Given these differences, it is likely that the type II binding mode has greater activating potential than the type I binding mode, a sobering possibility given that current clinical trials are dominated by type II RAF inhibitors.

Although most structures of dimeric BRAF do not show evidence of asymmetry that could explain the asymmetric allosteric coupling observed here, these structures were mostly determined with both protomers bound to αC-in inhibitors, likely negating any asymmetry present in apo or single drug-bound states. Interestingly, a recent cryo-EM structure^38^ of dimeric BRAF in complex with 14-3-3, determined in the absence of inhibitors, does show an asymmetric arrangement of the BRAF dimer with respect to the 14-3-3 dimer. This arrangement allows the tail of one protomer to reach into and inhibit the active site of the other, while the reciprocal interaction is prevented. Such dimer asymmetry may reflect an intrinsic regulatory mechanism of BRAF, as suggested by functional asymmetry observed in cells^35^, which αC-in inhibitors exploit to induce paradoxical activation. Consistent with this, we provide evidence that the protomers of the BRAF dimer are not homogeneously locked in the αC-in state, but rather exist in an equilibrium between locked and dynamic states. This lack of strict conformational constraints in the dimer provides an explanation for why substoichiometric binding of αC-in inhibitors strongly induces dimerization.

Despite the prominence of the V600E mutation in BRAF-driven cancers, the degree to which it destabilizes the αC-out state is unknown. By measuring the populations of the αC-in and αC-out states in both BRAF and BRAF^6000^, we have quantified the conformational effects of the V600E mutation for the first time. WT BRAF heavily favors the αC-out state, explaining its weak dimerization and low basal activity, and the conformational shift induced by the V600E mutation is subtle, with the kinase still sampling the αC-out state to ~50%. Consistent with this minimal effect, the mutation does not alter the asymmetric allosteric coupling between inhibitor binding and dimerization. Consequently, BRAF^V600E^ retains the ability to be paradoxically activated by αC-in inhibitors. This surprising observation is likely explained by the sheer magnitude of allosteric coupling unleashed by inhibitor binding, compared to which the relatively subtle effects of oncogenic mutations may be inconsequential. Given this, designing αC-in inhibitors that effectively differentiate between WT and V600E BRAF may require substantially dialing down the strength of the allosteric coupling between inhibitor binding and dimerization.

In summary, here we provide a quantitative model explaining how αC-in inhibitors induce paradoxical activation via asymmetric allosteric coupling between inhibitor binding and BRAF dimerization. The magnitude of this coupling appears to be unprecedented in the kinase inhibitor field and provides a striking contrast to the model of negative allostery proposed to explain activation by αC-out inhibitors. The asymmetric allosteric coupling mechanism allows type Il inhibitors to activate BRAF by nearly an order of magnitude. Susceptibility to these allosteric effects appears to be hard-wired into the kinase domain of BRAF but is likely to be tuned by other factors in the cell. In this light, it is interesting to consider the preclinical and clinical experience with type II RAF inhibitors. Several type II inhibitors studied here have either entered clinical trials for BRAF-driven cancers, like belvarafenib, or could be repurposed, like the FDA-approved chronic myeloid leukemia drug ponatinib. It is claimed that these molecules show minimal paradoxical activation compared to vemurafenib^27,28^. However, substantial paradoxical activation by these type II inhibitors has been observed in cell lines, particularly in cases with activating RAS mutations that confer a signaling environment conducive to RAF dimerization. For instance, ponatinib can activate ERK strongly in RAS-mutant cell lines, consistent with our observations, and at doses up to 1 μM^28^, substantially above the plasma level of 150 nM achievable in patients^39^. Similarly, while belvarafenib has shown promising activity in RAS-mutant cancers in preclinical and clinical studies, it has been observed to trigger an increase in MAPK signaling in some RAS-mutant cell lines^27^. Ultimately, whether paradoxical activation is observed with a particular inhibitor may depend on where the achievable dose lies with respect to the activation and inhibition sides of the paradoxical activation curve. Furthermore, resistance to both belvarafenib and another type II inhibitor naporafenib can be mediated by induction of homo- and heterodimers of the BRAF paralog ARAF^27,40^. ARAF binds both belvarafenib and naporafenib less potently than BRAF, presumably shifting the paradoxical activation curve into a higher concentration regime where activation instead of inhibition occurs. Taken together, these observations suggest that the asymmetric allosteric coupling uncovered here will continue to play a significant role in the treatment landscape of RAF-driven cancers.

## METHODS

### Protein expression and purification

Human BRAF kinase domain (residues 448–723 with a TEV protease cleavable N-terminal 6x-His tag in pProEX) containing 16 mutations to improve solubility^33^ was expressed in chemically competent BL21 (DE3) RIL *Escherichia coli* (Agilent) for 18 hours at 18° overnight. Following cell pellet sonication (Qsonica), lysates were clarified by centrifugation and loaded onto a HisTrap HP column (Cytiva). The column was washed with lysis buffer (50 mM Tris pH 8.0, 500 mM NaCl, 10% glycerol, 25 mM imidazole) and eluted with a 0–50% imidazole gradient over 12 column volumes using elution buffer (50 mM Tris pH 8.0, 500mMNaCl,10% glycerol, 500 mM imidazole). The His tag was cleaved overnight at 4°C with TEV protease. TEV protease was removed by an additional pass over a HisTrap HP column. Protein was further purified and desalted into desalt buffer (25 mM HEPES pH 7.5, 10% glycerol, 300 mM NaCl) using a Superdex S75 10/300 GL size exclusion column (Cytiva). Expression levels of BRAF mutants were increased by adding a SUMO tag N-terminal to the kinase domain.

Human MEK1 kinase domain (residues 1–393 with a TEV protease cleavable N-terminal 6x-His tag in pET-Duet) containing an inactivating K97R mutation was expressed in chemically competent BL21 (DE3) RIL *Escherichia coli* (Agilent) for 18 hours at 18° overnight. Cell pellets were sonicated in MEK lysis buffer (25 mM HEPES pH 7.5,500 mM NaCl, 10% glycerol, 10 mM BME, 1 mM PMSF, 1 X EDTA free protease inhibitors (Roche), 20 mM imidazole) and clarified by centrifugation. Lysate was loaded onto a HisTrap HP column (Cytiva) and washed with MEK lysis buffer before eluting with a 0–50% imidazole gradient over 12 column volumes using elution buffer (25 mM HEPES pH 7.5, 500mMNaCl,10% glycerol,10 mM BME, 300 mM imidazole). The His tag was cleaved overnight at 4°C with TEV protease. TEV protease was removed by an additional pass over the HisTrap HP column. Protein was further purified and desalted into desalt buffer (25 mM HEPES pH 7.5, 10% glycerol, 150mMNaCl) using a Superdex S75 10/300 GL size exclusion column (Cytiva).

### FRET experiments tracking BRAF dimerization

We used the K547C site on the αD-helix of BRAF to incorporate FRET dyes. Stopped-flow fluorescence anisotropy experiments showed that the labeling kinetics of BRAF^K547C^ were approximately two orders of magnitude faster than those of BRAF, indicating that the K547C site can be selectively labeled in the presence of the endogenous cysteine residues of BRAF. BRAF FRET samples were prepared by labeling two separate pools of BRAF at 25 μM on K547C with either donor (AF488 C_5_ maleimide, Thermo Fisher) or acceptor (AF568 C_5_ maleimide, Thermo Fisher) at 0.8:1 molar ratio of fluorophore to protein, on ice. Labeling reactions were quenched at 1 hour with 1 mM DTT. Donor-labeled and acceptor-labeled BRAF were then mixed at equal molar ratios and diluted into FRET buffer (25 mM HEPES pH 7.5, 300 mM NaCl, 10% glycerol, 10 mM MgCl_2_, 1 mM EGTA, 2% DMSO). BRAF FRET sensor (49 μL) was then added to 384-well inhibitor titration plates containing 1 μL of inhibitor in DMSO prepared using a mosquito liquid handling robot (ttp Labtech) and incubated at room temperature for 90 minutes to ensure the reactions were at equilibrium. Dimerization experiments consisted of inhibitor titrations containing 12 concentrations including a DMSO-only control. Experiments for each inhibitor were done in duplicate at six different BRAF concentrations. Fluorescence data were recorded with a custom-built fluorescence plate reader (Fluorescence Innovations) and contributions from the donor and acceptor fluorescence emission intensities (FRET_A/D_) were quantified by spectral unmixing using three basis functions for AF488 emission, AF568 emission, and Raman scattering^41^

### Global fitting and thermodynamic modeling of FRET data

FRET A/D values were globally fit to a thermodynamic model describing inhibitor-induced BRAF dimerization^34^ shown in Figure 1c using the fitting and simulation software KinTek Explorer. In this model B and D represent monomeric BRAF and drug/inhibitor, respectively, with B, BB, BBD, BBDD representing apo monomeric BRAF, apo dimeric BRAF, dimeric BRAF partially occupied with one inhibitor molecule, and dimeric BRAF fully saturated with two inhibitor molecules. The equilibrium dissociation constants ${K_{D}}^{\text{dimer}}$ and ${K_{D}}^{\text{drug}}$.describe BRAF dimerization and inhibitor binding to monomeric BRAF, respectively. Microscopic reversibility restricts the specific values and relationships between each equilibrium constant within a cyclic path, such that the product of equilibrium constants along a cycle must equal one^34,42^. Consequently, each reaction within the model can be described in terms of either ${K_{D}}^{\text{dimer}}$ or ${K_{D}}^{\text{drug}}$ together with the allosteric coupling parameters α and β, which quantify the allosteric coupling in the system as described in the main text.

**Table:** 

Reaction	Equilibrium dissociation constant	Definition of thermodynamic factors
B+B↔BB	$K_{D}^{dimer\;}$	
B+D↔BD	$K_{D}^{drug\;}$	
BB+D↔BBD	$K_{3}=\frac{1}{2}\alpha \cdot K_{D}^{drug\;}$	$\alpha =\frac{2\;\cdot \;K_{3}}{K_{D}^{drug\;}}$
BD+B↔BBD	$K_{4}=\frac{1}{2}\alpha \cdot K_{D}^{dimer\;}$	$\alpha =\frac{2\;\cdot \;K_{4}}{K_{D}^{dimer\;}}$
BD+BD↔BBDD	$K_{5}=\alpha \beta \cdot K_{D}^{dimer\;}$	$\beta =\frac{K_{5}}{2\;\cdot \;K_{4}}$
BBD+D↔BBDD	$K_{6}=2\beta \cdot K_{D}^{drug\;}$	$\beta =\frac{K_{6}}{2\;\cdot \;K_{D}^{drug}}$

The factor of two in the above definitions arises from the stoichiometry of the reactions and the presence of two inhibitor binding sites within the BRAF dimer^34^. Note that, as defined here, dimer promotion is represented by values of α and β less than 1. For convenience, in the main text we refer to the α and β values for the αC-in inhibitors as fold changes in affinity, i.e., an α factor of 1000 indicates 1000-fold increased binding or dimerization affinity.

The ratio of acceptor and donor emission intensity (A/D) measured from steady-state FRET was mapped onto the thermodynamic model using one fluorescence coefficient (c1) to describe monomeric forms of BRAF in solution (B, BD), and one fluorescence coefficient (c2) to describe dimeric forms of BRAF in solution (BB, BBD, BBDD).


A/D=c1×(B+BD)/(B+BD+BB+BD+BBDD)+c2×(BB+BBD+BBDD)/(B+BD+BB+BBD+BBDD)

In addition to the model parameters already mentioned (${K_{D}}^{\text{dimer}}$, ${K_{D}}^{\text{durg}}$, $\alpha {K_{D}}^{\text{dimer}}$, $\alpha \beta {K_{D}}^{\text{dimer}}$, $\alpha {K_{D}}^{\text{durg}},\;\beta {K_{D}}^{\text{durg}}$) BRAF concentrations for each experiment were included as floating parameters within the model (referred to as Bconc1-Bconc6). Analysis of error surfaces confirmed that these concentration parameters were well constrained in the fits (Supplementary Fig. 1). The relatively weak dimerization affinity of apo BRAF (${K_{D}}^{\text{dimer}}$) was determined in separate experiments using high concentrations of BRAF to maximize the dimerization signal. This value was then used to constrain ${K_{D}}^{\text{dimer}}$ dimer in the fitting of other datasets, allowing lower concentrations of BRAF to be used. Under these conditions, intermolecular FRET data yielded constrained values for all other parameters within the model as determined by onedimensional error surface analysis using chi^2^ thresholds calculated as previously described (Supplementary Fig. 1 and Supplementary Fig. 3)^43^. Two-dimensional error surface analysis confirmed that the pairs of equilibrium constants from which the α and β factors were derived (e.g. ${K_{D}}^{\text{dimer}}$ and $\alpha {K_{D}}^{\text{dimer}}$) are well constrained with respect to one another (Supplementary Fig. 3c).

### Kinase activity assays

Kinase activity of BRAF was measured using the FRET-based LanthaScreen kinase activity assay (Thermo Fischer). Kinase dead MEK1 K97R was labeled at 40 μM with Alexa Fluor 488 C_5_ maleimide at a 1:1 molar ratio on ice for 60 minutes. The labeling reaction was quenched using 1 mM DTT and desalted into 25m MH EPES pH 7.5, 10% glycerol, and 150 mM NaCl. BRAF^15m^(BRAF^16m^ with the additional E667F reversion mutation that restores MEK binding) was incubated at 400 nM with 2 μM MEK and 2x kinase buffer (50 mM HEPES pH 7.5, 0.2 mg/mL bovine γ-Globulins, 20 mM MgCl, 600 mM NaCl, 2mM EGTA) for 15 minutes. Inhibitor (1 μL) in 50% DMSO was then added to the BRAF/MEK reaction and incubated at room temperature for 60 minutes. The kinase reaction was then initiated with the addition of 250 μM ATP for a final reaction concentration of 200 nM BRAF, 1 μM MEK, 100 μM ATP, 1X kinase buffer, and 5% DMSO with inhibitor and incubated for 60 minutes. Reactions were quenched with a 2X dilution into TR-FRET buffer (Thermo Fischer) with 40 mM EDTA and 4 nM LanthaScreen Tb-pMAP2K1 (pSer 217/pSer 221) antibody (Thermo Fischer) and incubated at room temperature for two hours. The TR-FRET ratio was measured using a Tecan M1000 pro plate reader with excitation at 340 nm followed by a 100 μs delay before reading emission at 490 nm (donor) and 520 nm (acceptor) with a 200 μs integration time. Increases in kinase activity were inferred from increases in FRET (acceptor/donor ratio). Kinase turnover (s^−1^) was interpolated from a phoshoMEK1 standard curve. Kinase activity of BRAF^V600E^ was measured using the same protocol except for using a lower kinase concentration (20 nM) and shorter incubation time (15 min) to account for the greater kinase activity of the mutant. Turnover values were in good agreement with previously published values^44^.

### DEER spectroscopy

BRAF DEER samples were prepared by labeling 10 μM BRAF containing three dimer-breaking mutations (BRAF^DB^, R509H, L515G, M517W)^45^ on the αC-helix (Q493C) and the αG-helix (Q664C) with a 2-fold excess of 4-Maleimido-TEMPO for 45 minutes at 4°C. Spin-labeled BRAF was concentrated to 60–80 μM, buffered in D_2_O with 25 mM HEPESpH7.5, 500 mM NaCl, and 10% d_8_-glycerol and rabidly froze in 1.1 mm ID/1.6 mm OD quartz capillary tubes using liquid nitrogen-cooled isopropanol. For samples containing inhibitors, prior to freezing, BRAF was incubated for 90 minutes with a 5-fold molar excess of inhibitor dissolved in deuterated DMSO. DEER spectra were collected at 65K on an Elexsys E580 spectrometer (Bruker) equipped with an EN5107 resonator operating at Q-band frequencies using parameters previously described. ^46^ Data were analyzed using custom software (github.com/thompsar/Venison) written in python and based on DeerAnalysis 2017. DEER data were phased and background corrected using a homogenous background model to derive the DEER waveform. Distance distributions were obtained by fitting these waveforms using unconstrained Tikhonov regularization, with smoothing parameter λ chosen using the L-curve method and leave-one-out cross-validation. Features of the DEER waveform that contributed to unstable populations that were distinct from the primary populations and beyond the sensitivity limit of the 6 μs evolution time (~60 Å) were suppressed by incorporation into the background model. The distribution obtained by Tikhonov regularization using this corrected waveform was used to initialize fitting of the waveforms to a sum of Gaussians model that describes the centers of the spin-spin distances, as well as the widths and mole fractions. The number of subpopulations was determined by selecting the fewest number of Gaussian centers that met the RMSD minimization threshold calculated by the Bayesian information criterion. Confidence intervals were calculated with 5,000 Monte Carlo simulations of Gaussian fits to the background-corrected waveforms. All uncertainties quoted in main text and figures represent 75% confidence intervals.

Predicted spin-spin distance distributions for 4-maleimido-TEMPO were calculated from x-ray structures using the mtssIWizard^47^ function withing mtsslSuite (www.mtsslsuite.isb.ukbonn.de) with the tight conformational search setting for generating spin probe ensembles. Calculated distance distributions agreed well with experiment allowing unambiguous assignment of peaks in the experimental distance distributions to individual structural states (Supplementary Fig. 13).

### ^19^F NMR spectroscopy

BRAF ^19^F samples were prepared by labeling 50 μM of BRAF^16m^ on a single αC-helix cysteine (Q493C) with a 1.75 molar excess of 3-bromo-1, 1, 1-trifluoroacetone (BTFA) for one hour at 4°C. Samples were quenched with 1 mM DTT and desalted into NMR buffer (25 mM HEPES pH 7.5, 500 mM NaCl, 10% glycerol) supplemented with 10% D_2_O and 0.005% trifluoroacetic acid as an internal reference.

^19^F NMR experiments were performed at 298 K using a Bruker 600 MHz Avance NEO equipped with a 5 mm cryogenic triple resonance probe tuned to 565.123 MHz. 1D spectra were collected using the zg pulse program (Bruker TopSpin 4.1.4) with a 13.5 μs 90° pulse time, 0.2 s acquisition time, and a 1 s D1 relaxation delay time. Transverse relaxation $\left.{(T}_{2}\right)$ experiments were performed using the Carr-Purcell-Meiboom-Gill (CPMG) pulse sequence with a 12 μs 90° pulse time, 2 s D1 relaxation delay, 200 μs D20 fixed spin-echo time, and a 24 μs 180° refocusing period. Spectra were acquired with 2048 scans with total transverse magnetization times of 0.4,1.6,2.4,3.2,6.4,12.8,25.6,51.2 and 102.4 ms. 1D spectra were processed using MestReNova 14.3.0 by aligning the TFA reference to −75.32ppm, applying automatic zeroth and first-order phase corrections, a 3-degree polynomial Bernstein baseline correction, and 1 Hz line broadening correction.

$T_{2}$ relaxation profiles were created by measuring the intensity at −84.42 ppm (αC-out) and −84.29ppm (αC-in) as a function of delay time and fit to both single and double exponential decay models in GraphPad Prism 9.4.0. with the double exponential being the preferred model for both resonances (pa<0.0001). In a separate $T_{2}$ analysis, each spectrum was subjected to spectral deconvolution using OriginPro 2022 by fitting the 1D spectra to a three-component Lorentzian model. The time dependence of the component amplitudes is shown in Figure 4b.

### Mass spectrometry analysis of commercial kinase inhibitors

Commercially available RAF kinase inhibitors were purchased from Selleckchem and TargetMol. High resolution mass spectrometry data were collected on a Bruker BioTOF II instrument with an infusion electrospray ionizer. Compounds were dissolved in DMSO to a concentration of 10 mM. Stocks were diluted 100x with MeOH and injected at a rate of 10 μL/min. Mass spectrometry was run and analyzed in positive-ion mode with either a PEG 600 or PEG 400 internal standard. Data were analyzed using Bruker Data Analysis Software.

## Supplementary Material

Supplement 1
