# Supplementary material for: Allosteric coupling asymmetry mediates paradoxical activation of BRAF
Source: bioRxiv. 2023 Apr 19:2023.04.18.536450. Preprint. [Version 1] doi: 10.1101/2023.04.18.536450 (PMC10153139; doi:10.1101/2023.04.18.536450)
Supplement: Supplement 1 [file media-1.pdf]

Supplementary information

Allosteric coupling asymmetry mediates paradoxical activation of BRAF

Damien M. Rasmussen[1], Manny M. Semonis[1], Joseph M. Muretta[2], Andrew R. Thompson[2], David D. Thomas[2], William. Pomerantz[3], Nicholas M. Levinson[1]

[1]Department of Pharmacology, University of Minnesota, Minneapolis, MN, 55455

[2]Department of Biochemistry, Molecular Biology, and Biophysics, University of Minnesota, Minneapolis, MN, 55455

[3]Department of Chemistry, University of Minnesota, Minneapolis, MN, 55455

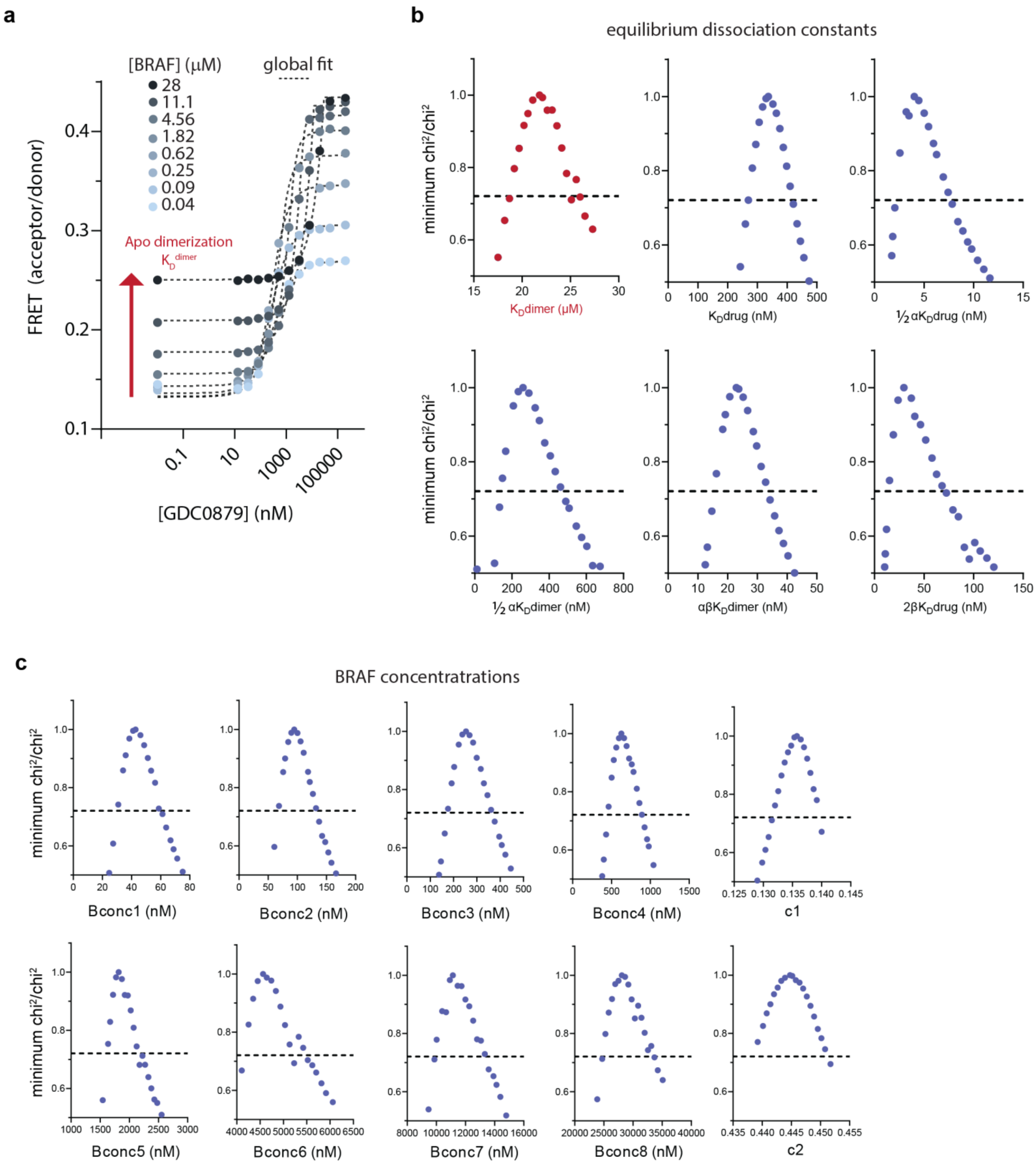

Supplementary Figure 1. Quantifying apo BRAF dimerization affinity (K_D^{dimer}). a) Intermolecular FRET experiments tracking BRAF dimerization as a function of GDC0879 concentration were carried out at high BRAF concentrations to help define the relatively weak apo BRAF dimerization affinity. Black dotted lines represent global fits to the thermodynamic model shown in Figure 1c (see Methods). The red arrow highlights apo BRAF dimerization (K_D^{dimer}) occurring with no inhibitor present. **b,c)** Representative one-dimensional error surface analysis of the global fit parameters for the FRET data shown in panel a, including equilibrium dissociation constants (**b**) and BRAF concentrations (**c**). The black dotted line represents the chi^2 threshold used to establish 95% CIs for each parameter (see Methods). Note that all parameters shown are well-constrained within this limit as indicated by their intersection with the chi^2 threshold boundary.

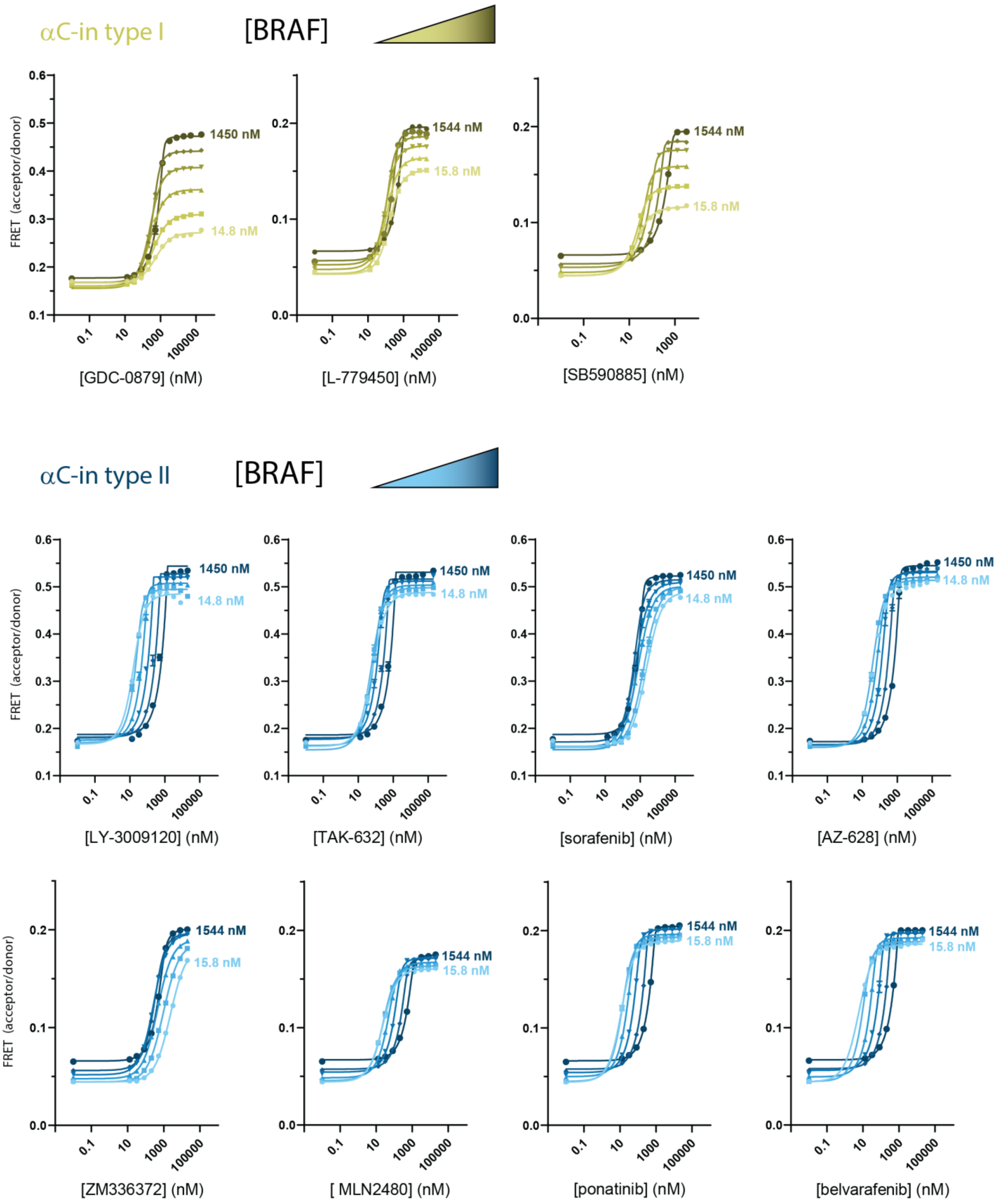

αC-in type I
[BRAF]
FRET (acceptor/donor)
1450 nM
14.8 nM
[GDC-0879] (nM)
1544 nM
15.8 nM
[L-779450] (nM)
1544 nM
15.8 nM
[SB590885] (nM)
αC-in type II
[BRAF]
1450 nM
14.8 nM
[LY-3009120] (nM)
1450 nM
14.8 nM
[TAK-632] (nM)
1450 nM
14.8 nM
[sorafenib] (nM)
1450 nM
14.8 nM
[AZ-628] (nM)
1544 nM
15.8 nM
[ZM336372] (nM)
1544 nM
15.8 nM
[MLN2480] (nM)
1544 nM
15.8 nM
[ponatinib] (nM)
1544 nM
15.8 nM
[belvarafenib] (nM)

Supplementary Figure 2. Intermolecular FRET experiments measuring inhibitor-induced BRAF dimerization. Representative data for all αC-in inhibitors showing BRAF dimerization for type I (yellow) and type II (blue) inhibitors. Gradients from light to dark represent experiments done at increasing BRAF concentrations. Lines are fits of individual titration data sets to a quadratic binding model.

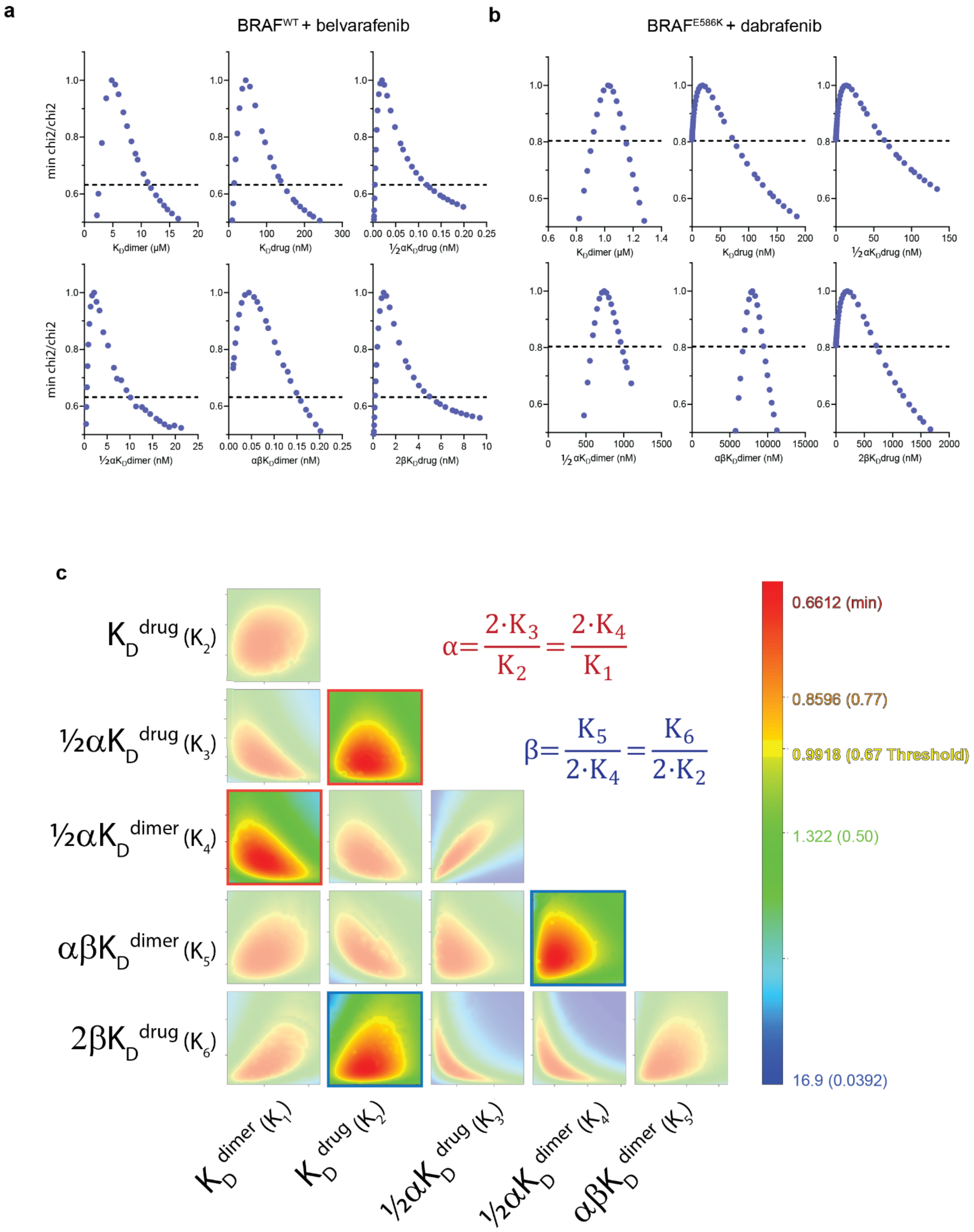

a
BRAF^{WT} + belvarafenib
b
BRAF^{E586K} + dabrafenib
min chi2/chi2
$K_D$dimer (µM)
$K_D$drug (nM)
½α$K_D$drug (nM)
½α$K_D$dimer (nM)
αβ$K_D$dimer (nM)
2β$K_D$drug (nM)
c
K_D^{drug} (K_2)
½αK_D^{drug} (K_3)
½αK_D^{dimer} (K_4)
αβK_D^{dimer} (K_5)
2βK_D^{drug} (K_6)
K_D^{dimer} (K_1)
$\alpha = \frac{2 \cdot K_3}{K_2} = \frac{2 \cdot K_4}{K_1}$
$\beta = \frac{K_5}{2 \cdot K_4} = \frac{K_6}{2 \cdot K_2}$
0.6612 (min)
0.8596 (0.77)
0.9918 (0.67 Threshold)
1.322 (0.50)
16.9 (0.0392)

Supplementary Figure 3. One- and two-dimensional error surfaces show well-constrained parameters. One-dimensional error surface analysis of the global fit parameters from FRET experiments with the αC-in type II inhibitor belvarafenib (**a**) and the αC-out inhibitor dabrafenib (**b**)**.** The black dotted line represents the chi^2 threshold used to establish 95% CIs for each parameter (see Methods). Note that all parameters shown are well-constrained within this limit as indicated by their intersection with the chi^2 threshold boundary. **c,** A representative two-dimensional confidence contour analysis of parameters from the FRET experiments with BRAF and GDC0879 shown in Supplementary Figure 1a performed in KinTek Explorer. All possible pair-wise combinations of unconstrained parameters within the model were systematically varied to test for the presence of well constrained chi^2 minima and examine covariance between parameters. The color bar on the right shows the absolute chi^2 value with red representing the overall best-fit and yellow representing the boundary at the chi^2 threshold for 95% CIs. This analysis shows that all parameters in the model are well-constrained by the data. Furthermore, the allosteric coupling factors α and β defined by the ratios of specific equilibrium dissociation constants (see Methods) are well-constrained within the defined boundaries.

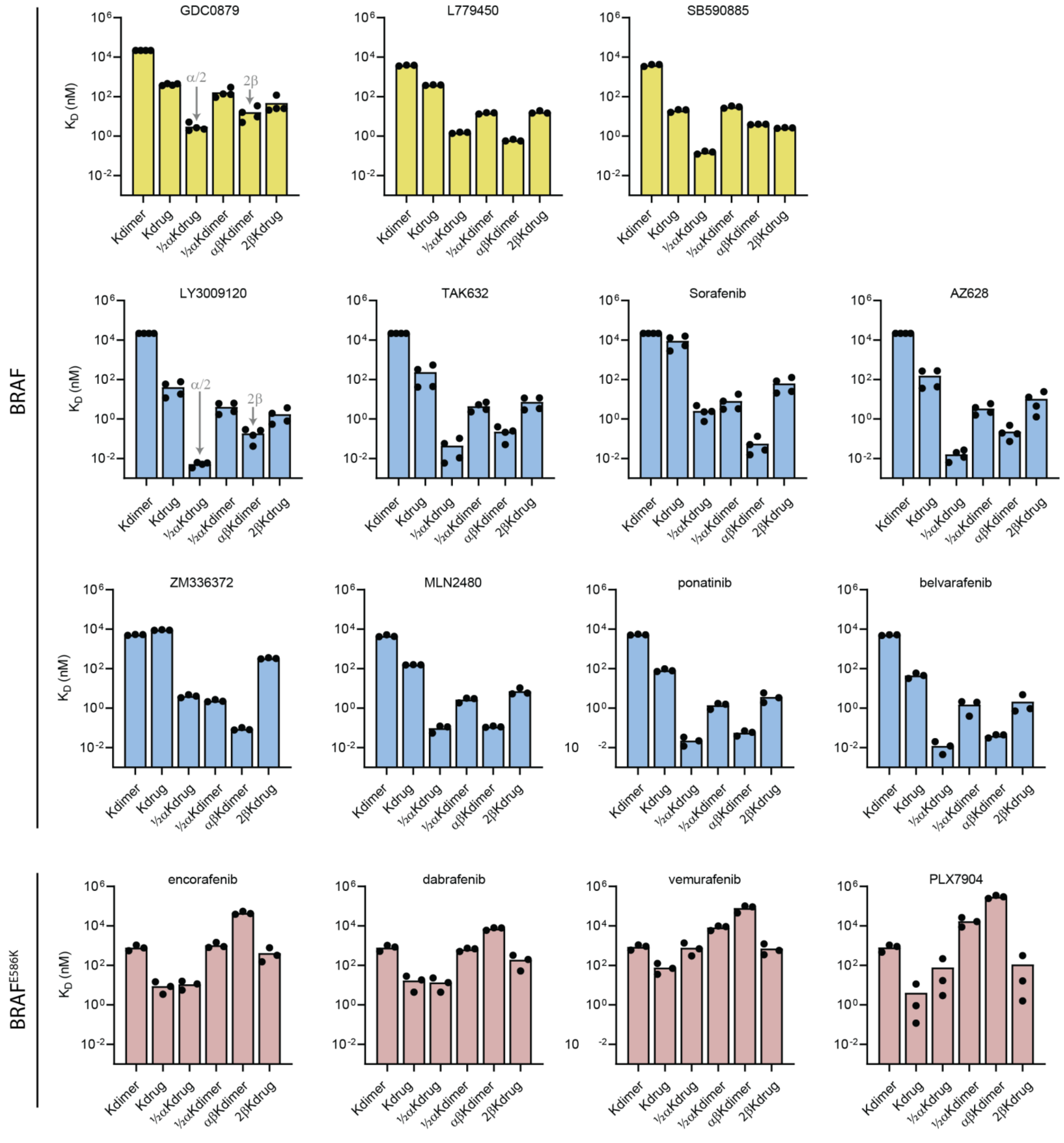

Supplementary Figure 4. Equilibrium dissociation constants derived from global fitting of FRET data for all inhibitors. Equilibrium dissociation constants derived from the global fitting of the intermolecular FRET dimerization data to the thermodynamic model describing the allosteric coupling between inhibitor binding and BRAF dimerization. αC-in type I (yellow) and αC-in type II (blue) show a positive allosteric coupling and increase BRAF dimerization whereas αC-out inhibitors (red) show a negative allosteric coupling and weaken BRAF^{E586K} dimerization. Data represent best-fit values from global analysis of n=4 independent experiments done in duplicate.

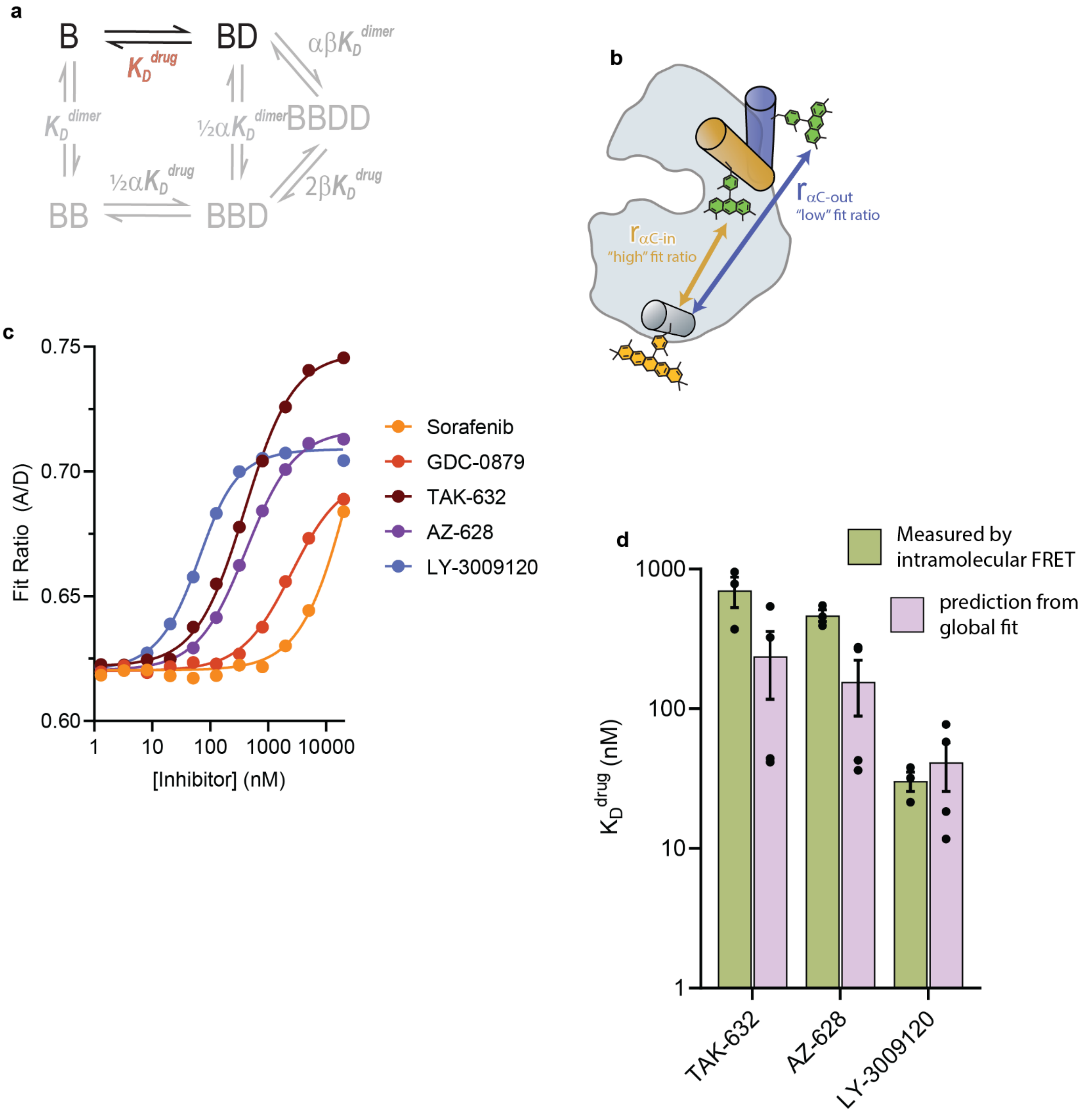

Supplementary Figure 5. Inhibitor affinities for monomeric BRAF (K_D^{drug}) measured by intramolecular FRET. a) Schematic highlighting the equilibrium dissociation constant that is being independently measured in the context of the thermodynamic model used for global fitting. **b)** Schematic showing the intramolecular FRET biosensor used to quantify inhibitor binding to monomeric BRAF containing dimer disrupting mutations. Inhibitor-induced changes in the conformation of the αC-helix upon binding leads to a change in FRET. **c)** Inhibitor binding to monomeric BRAF as indicated by changes in FRET (acceptor / donor ratio). Binding curves were fit to a quadratic binding model in GraphPad Prism to extract K_D^{drug}. **d)** Best-fit values for K_D^{drug} measured directly by intramolecular FRET (green) in panel c compared to best-fit values predicted from the global fitting analysis of intermolecular FRET dimerization data (pink). Data represents the mean ± s.e.m.; n=3 independent experiments.

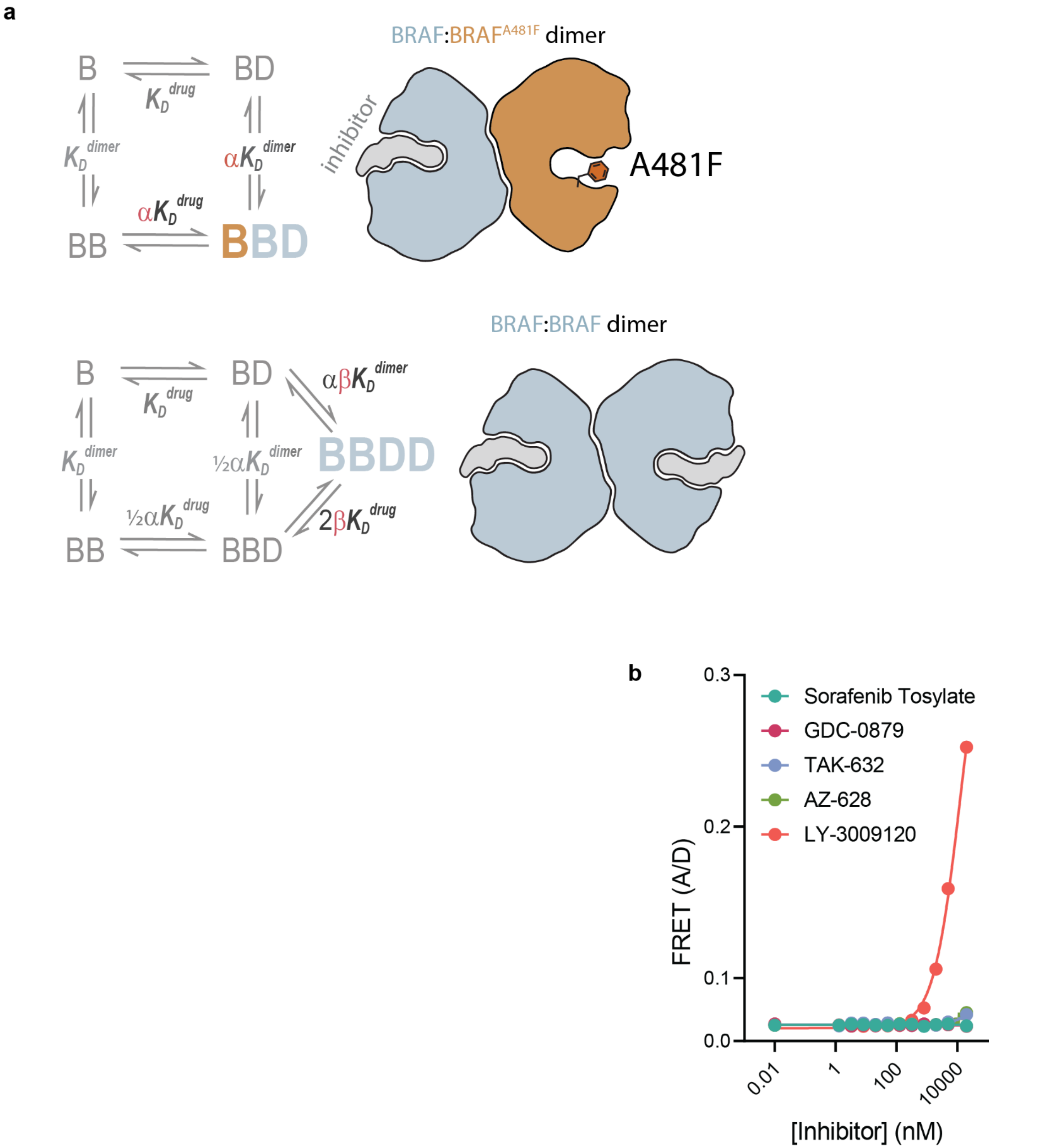

Supplementary Figure 6. The A481F active site mutation blocks inhibitor binding and prevents inhibitor-induced dimerization. a) A schematic demonstrating how the A481F eliminates the effects of β on BRAF dimerization by preventing the formation of BRAF dimers with both active sites occupied by inhibitors. **b)** Intermolecular FRET dimerization data showing the formation of $BRAF^{A481F}$ / $BRAF^{A481F}$ heterodimers as a function of αC-in inhibitor concentration. Note that except for LY3009120, αC-in inhibitors fail to induce A481F homodimers.

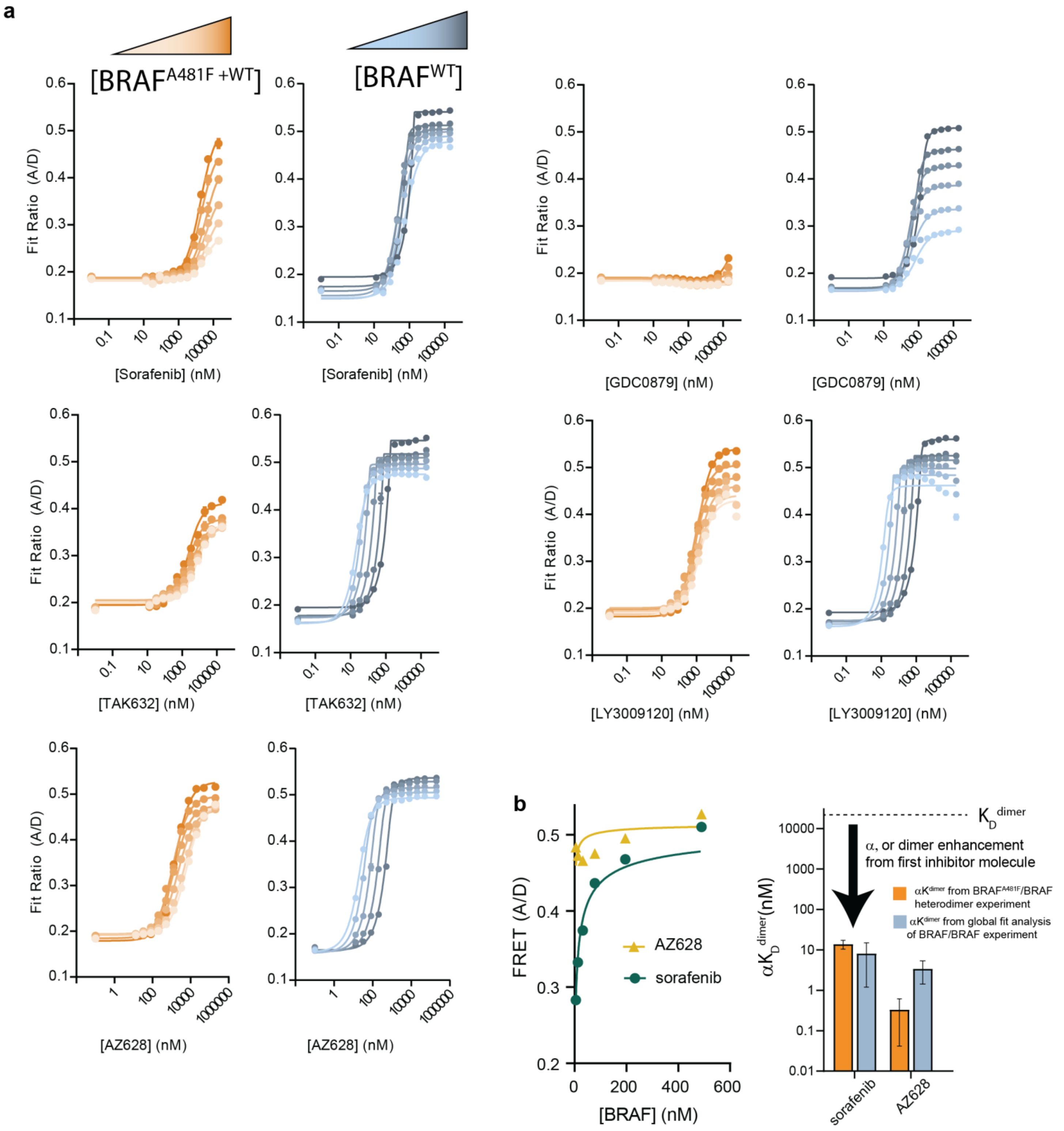

Supplementary Figure 7. The binding of a single αC-in type II molecule is sufficient to dramatically increase BRAF dimerization affinity. a) Representative intermolecular FRET dimerization data showing the formation of BRAF^{A481F}/BRAF heterodimers (orange) and BRAF/BRAF homodimers (blue) as a function of αC-in inhibitor concentration. With the exception if GDC0879, all αC-in type II inhibitors induce significant formation of BRAF^{A481F}/BRAF heterodimers. **b)** The upper baseline at saturating inhibitor concentrations in panel a were fit to a monomer-dimer equilibrium model. These affinities agree well with αK_D^{dimer} values calculated from the global fitting analysis of independent FRET experiments.

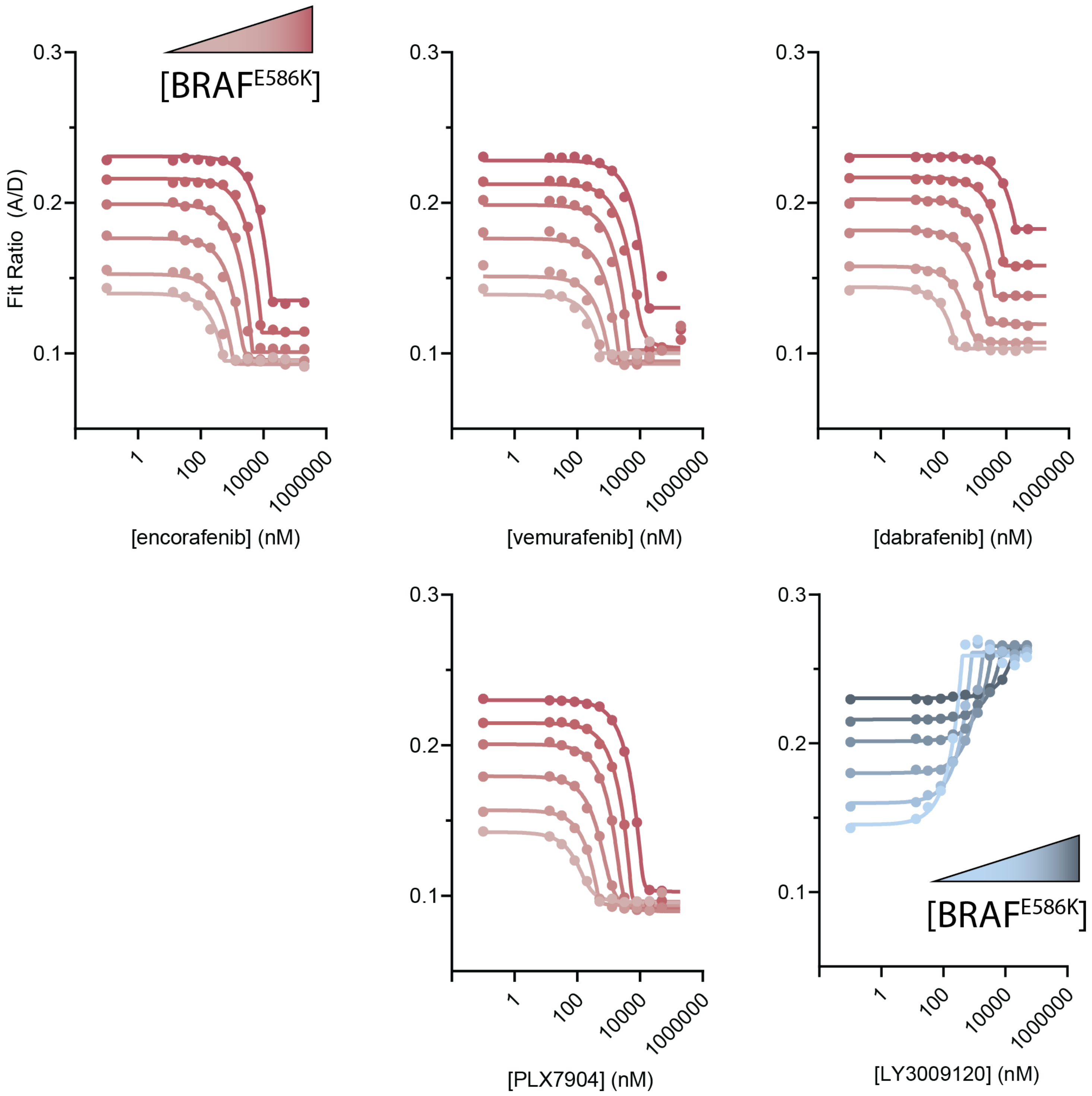

Supplementary Figure 8. Intermolecular FRET experiments measuring the disruption of BRAF^{E586K} dimerization by αC-out inhibitors. Representative data showing BRAF^{E586K} dimerization as a function of αC-out inhibitors (red) and the αC-in inhibitor LY3009120 for comparison. Gradients from light to dark represent experiments done at increasing BRAF concentration.

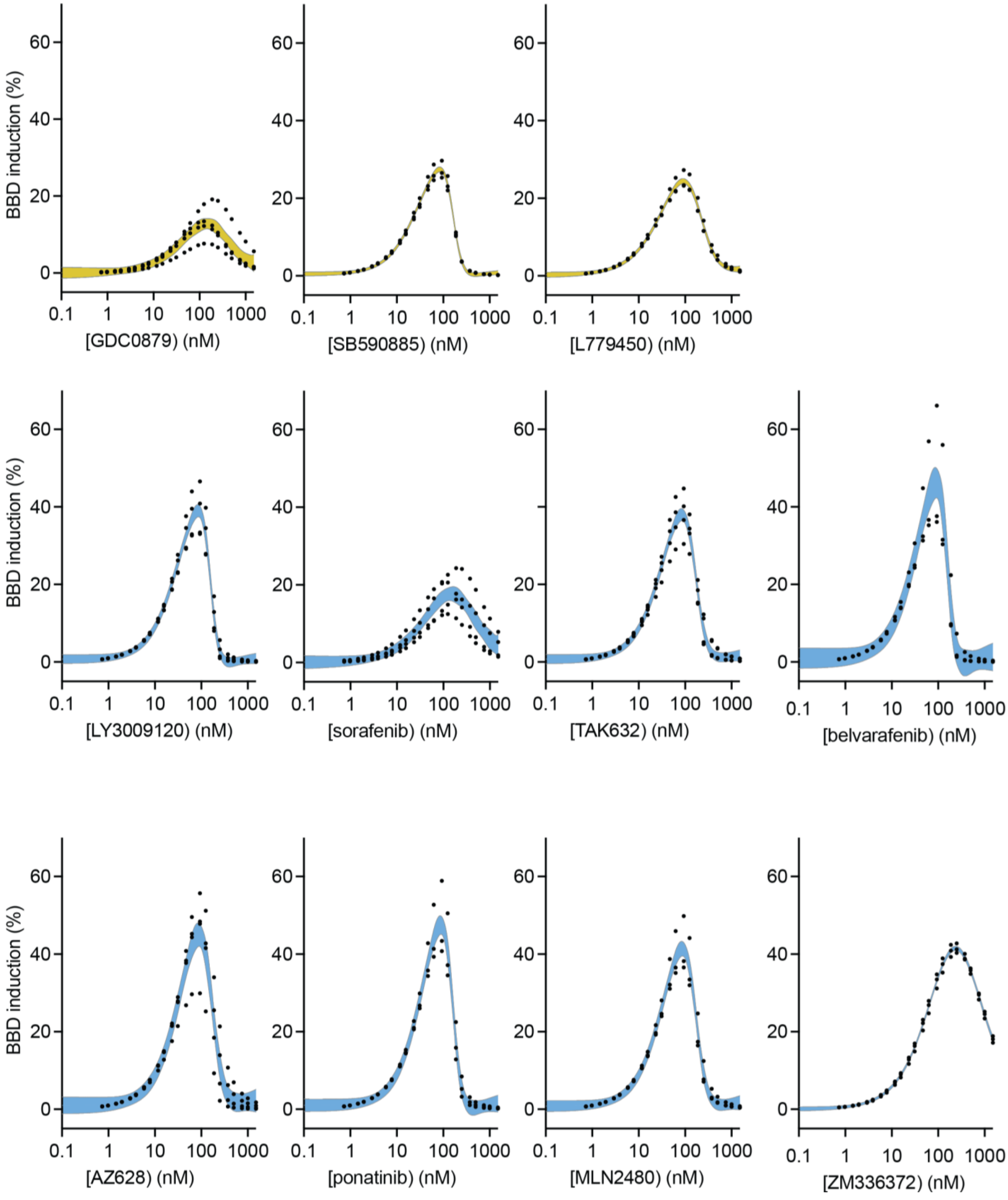
BBD induction (%)
60
40
20
0
0.1
1
10
100
1000
[GDC0879) (nM)
[SB590885) (nM)
[L779450) (nM)
[LY3009120) (nM)
[sorafenib) (nM)
[TAK632) (nM)
[belvarafenib) (nM)
[AZ628) (nM)
[ponatinib) (nM)
[MLN2480) (nM)
[ZM336372) (nM)

Supplementary Figure 9. Simulations of partially occupied BRAF dimer formation. The formation of partially occupied "*BBD*" BRAF dimers was simulated as a function of inhibitor concentration using the thermodynamic model (Figure 1c) parameterized for each inhibitor via the global analysis of intermolecular FRET data (see Methods). Multiple independent simulations (black dots) were performed for each inhibitor based on models parameterized with separate FRET datasets. These combined simulations were fit to a bell-shaped dose-response curve as shown for αC-in type I (yellow) and αC-in type II (blue) inhibitors. The 95% CI of the fit model for each simulation is represented as a colored ribbon. Data represent simulated values from best-fit parameters; n=3 independent experiments performed in duplicate.

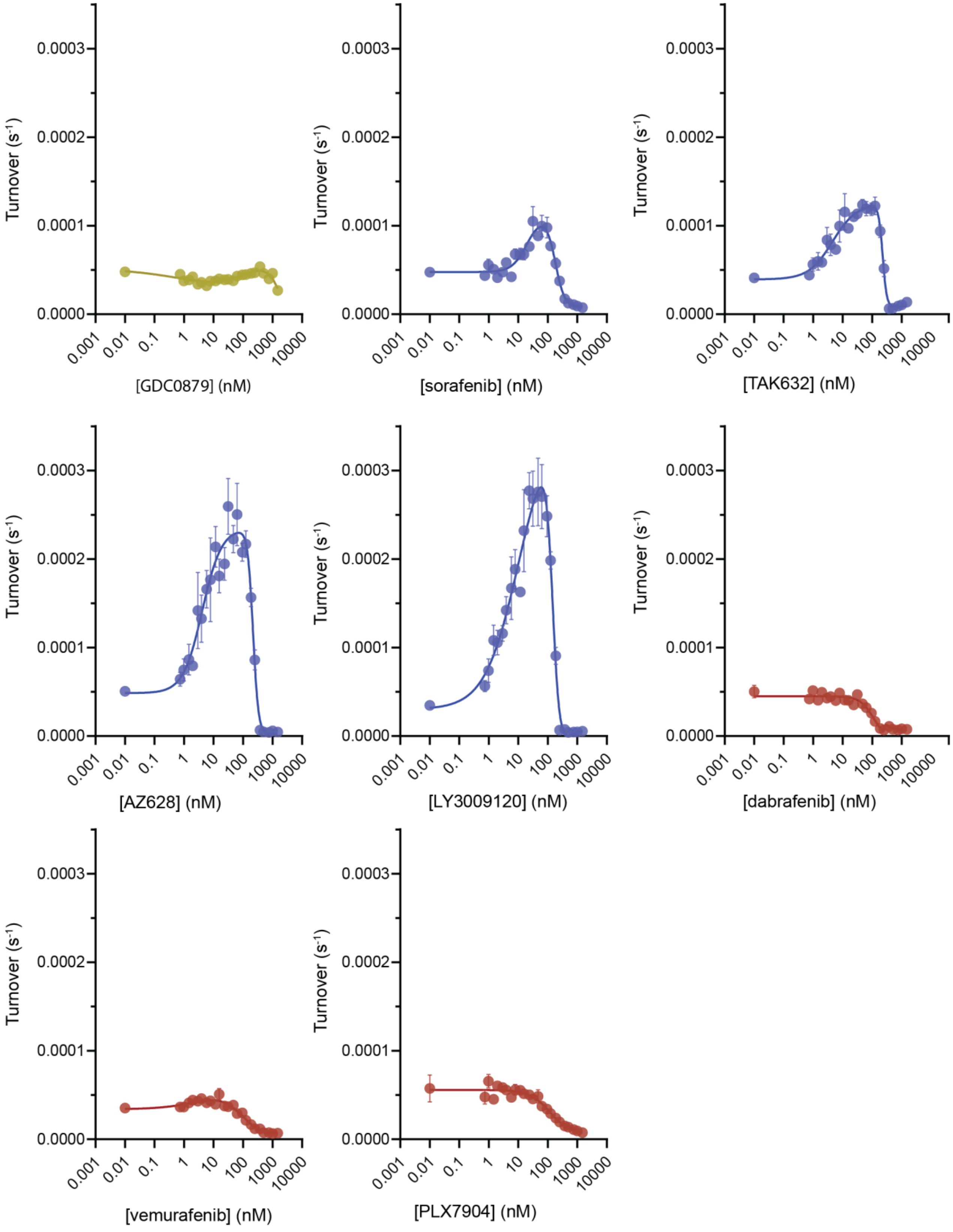

Supplementary Figure 10. αC-in inhibitors produce a dose-dependent increase in BRAF kinase activity. Phosphorylation of MEK by BRAF was measured as a function of αC-in type I (yellow), αC-in type II (blue), and αC-out (red) inhibitors. The overall activation potential of αC-in type II inhibitors is far greater than the αC-in type I inhibitor GDC0879, in agreement with model predictions shown in Figure 2e. Data were fit to a bell-shaped dose-response curve (solid lines) in GraphPad Prism and represent the mean ± s.e.m.; n=3 independent experiments performed in duplicate.

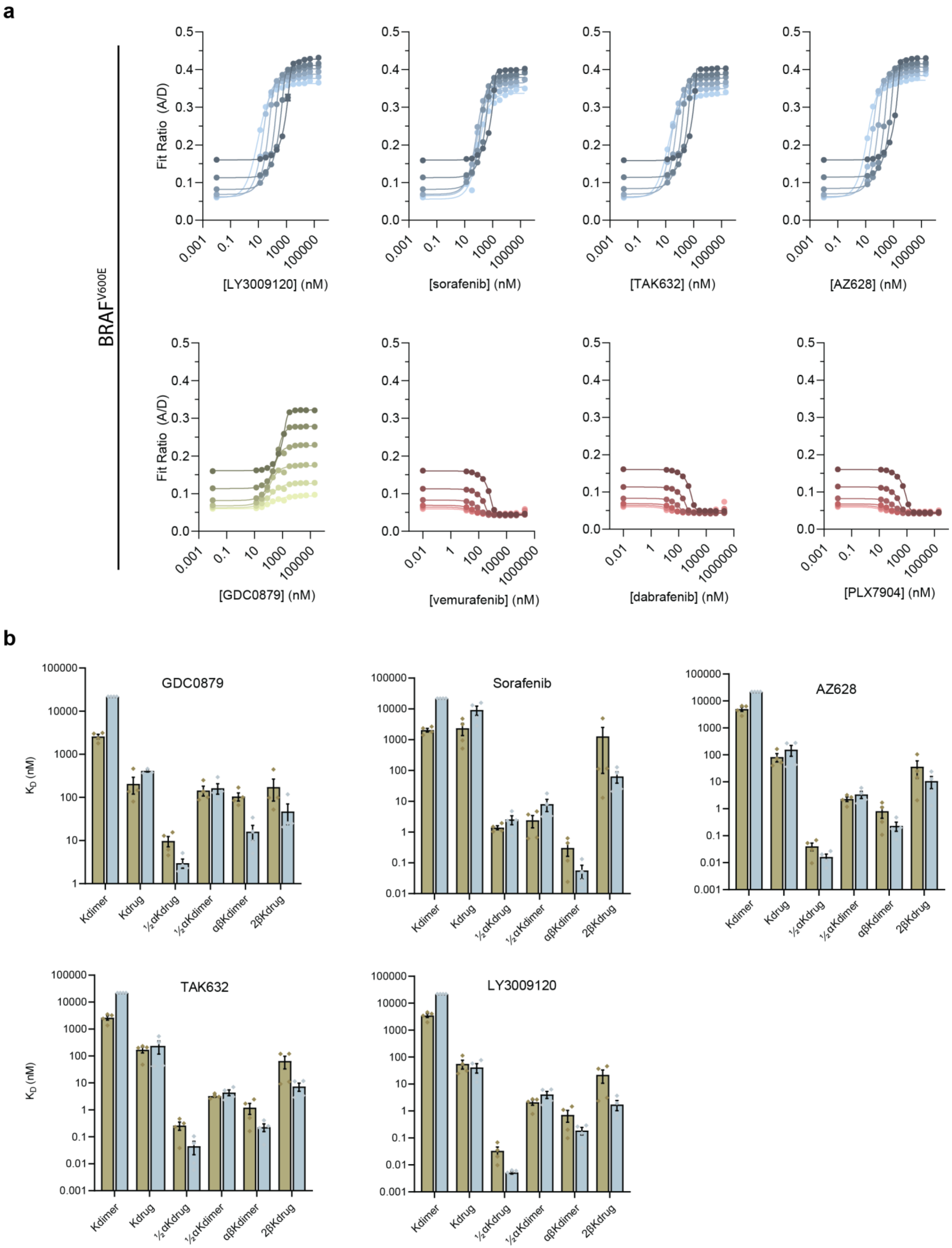
a
b
BRAF^V600E
Fit Ratio (A/D)
[LY3009120] (nM)
[sorafenib] (nM)
[TAK632] (nM)
[AZ628] (nM)
[GDC0879] (nM)
[vemurafenib] (nM)
[dabrafenib] (nM)
[PLX7904] (nM)
K_D (nM)
GDC0879
Sorafenib
AZ628
TAK632
LY3009120
Kdimer
Kdrug
½αKdrug
½αKdimer
αβKdimer
2βKdrug

Supplementary Figure 11. The V600E mutation does not substantially influence the asymmetric allosteric coupling between αC-in inhibitors and BRAF dimerization. a) Representative data showing $BRAF^{V600E}$ dimerization as a function of αC-in type II (blue), αC-in type I (yellow) and αC-out (red) inhibitors. Gradients from light to dark represent experiments done at increasing BRAF concentration. **b)** Equilibrium dissociation constants for $BRAF^{V600E}$ (brown) and BRAF (blue) determined from the global fitting analysis of intermolecular FRET experiments. Data represent mean ± s.e.m.; n=4 independent experiments done in duplicate.

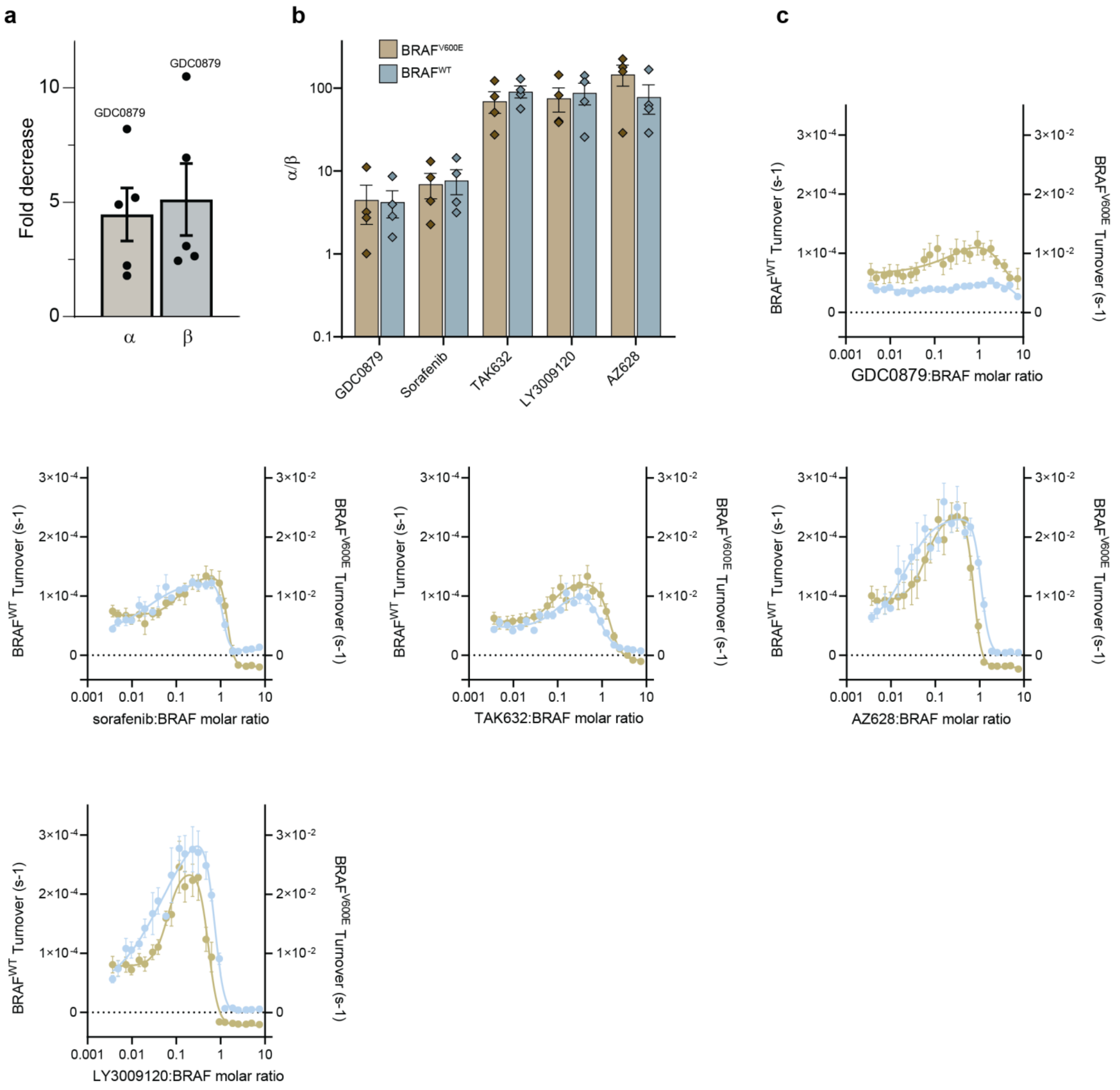

Supplementary Figure 12. αC-in inhibitors maintain the ability to induce increases in BRAF^{V600E} activity. a) Fold decrease of the allostric coupling parameters α and β from BRAF to BRAF^{V600E}. Fold decreases for each αC-in inhibitor are shown as black dots. Both α and β are decreased by nearly equivalent amounts for all αC-in inhibitors. Data represent mean ± s.e.m.; n=5 independent experiments done in quadruplicate. **b)** Allosteric coupling ratios (α/β) for BRAF^{V600E} (brown) and BRAF (blue) from intermolecular FRET experiments with αC-in inhibitors. Ratios were calculated using dissociation constants derived from the global fitting analysis of FRET data (see Methods). Despite the fold changes shown in **a**, the allosteric coupling ratios (α/β) remain nearly identical between BRAF^{V600E} and BRAF. Data represent the mean ± s.e.m.; n=4 independent experiments done in duplicate. **c)** Overlay of BRAF^{V600E} (brown) and BRAF (blue) kinase activity as a function inhibitor concentration. Data represent mean ± s.e.m.; n=3 independent experiments done in duplicate.

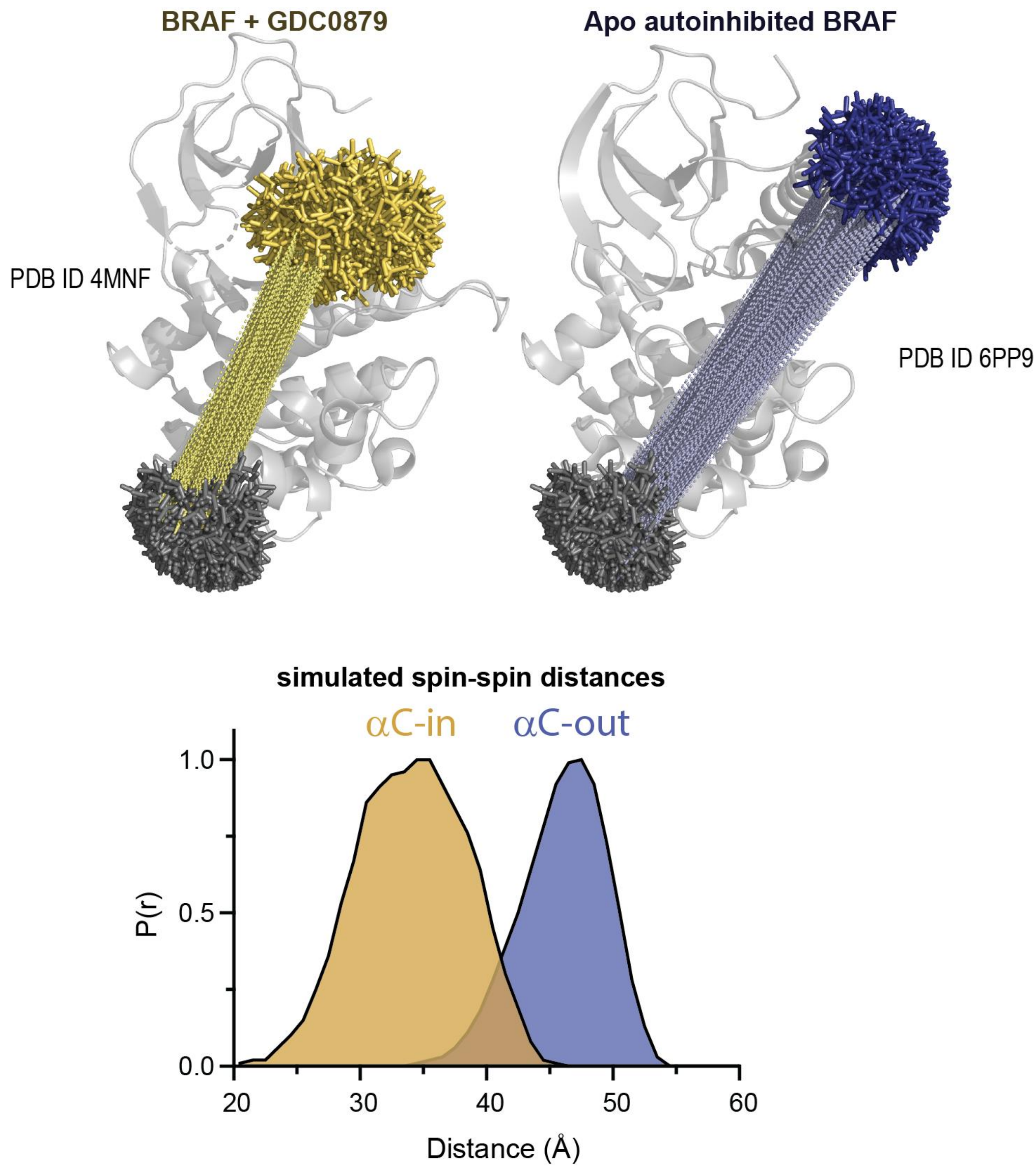

Supplementary Figure 13. Gaussian fits of the DEER data can be assigned to structural states of BRAF. MtsslWizard was used to generate spin-spin distance distributions using BRAF crystal structures. Rotamers of 4-maleimido-TEMPO were generated from a GDC0879 bound BRAF structure adopting an αC-in state (yellow) and a structure of apo BRAF adopting an αC-out state (blue). Individual spin-spin distances (dashed lines) were calculated and compiled to create spin-spin distance distributions used to assign the Gaussian distance distributions from DEER experiments measuring the αC-helix represented in Figure 3f. (see Methods).

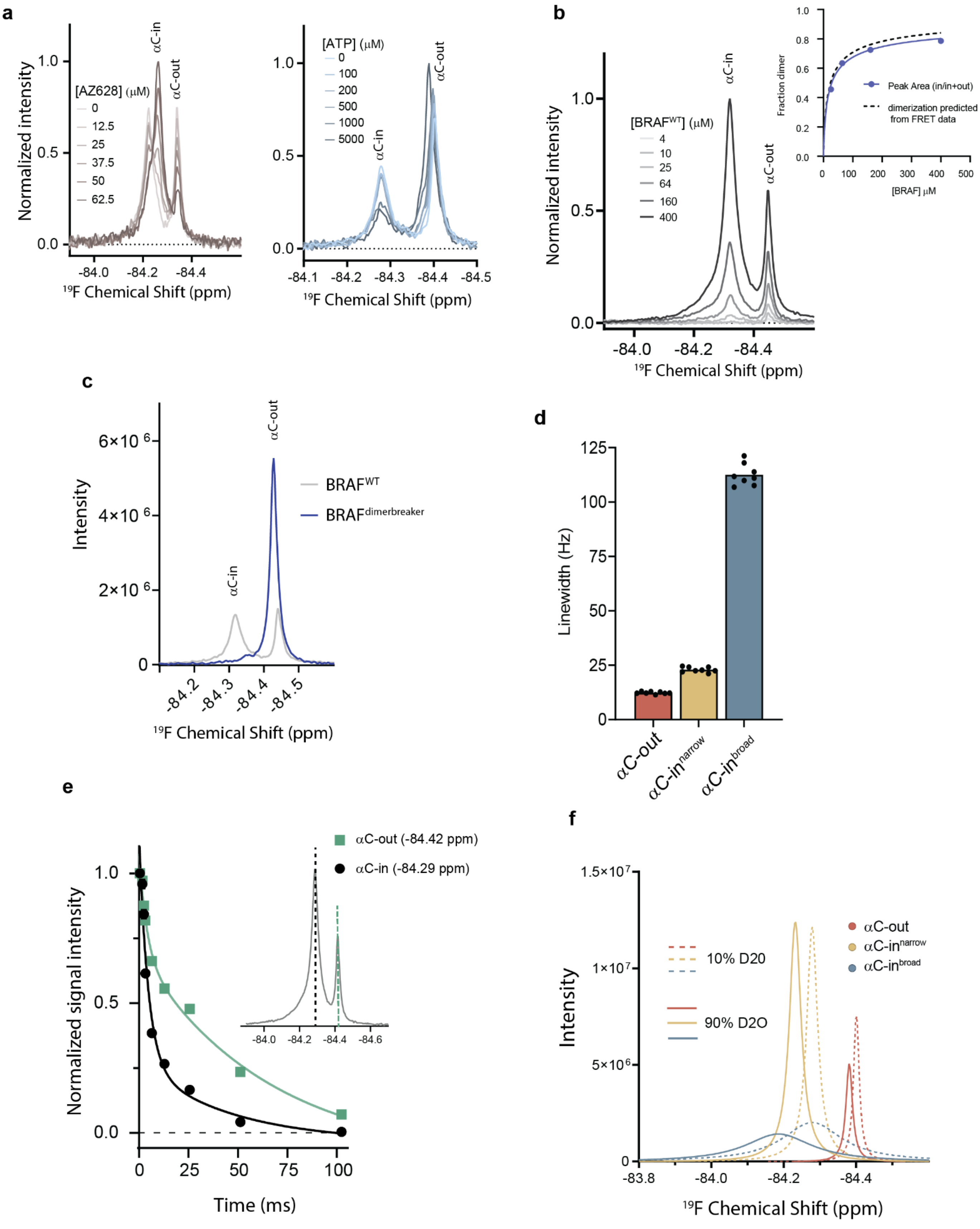
a
Normalized intensity
[AZ628] (µM)
0
12.5
25
37.5
50
62.5
αC-in
αC-out
¹⁹F Chemical Shift (ppm)
[ATP] (µM)
0
100
200
500
1000
5000
b
[BRAF^{WT}] (µM)
4
10
25
64
160
400
Fraction dimer
Peak Area (in/in+out)
dimerization predicted from FRET data
[BRAF] µM
c
Intensity
BRAF^{WT}
BRAF^{dimerbreaker}
d
Linewidth (Hz)
αC-out
αC-in^{narrow}
αC-in^{broad}
e
Normalized signal intensity
αC-out (-84.42 ppm)
αC-in (-84.29 ppm)
Time (ms)
f
Intensity
10% D20
90% D2O
αC-out
αC-in^{narrow}
αC-in^{broad}
¹⁹F Chemical Shift (ppm)

Supplementary Figure 14. ^{19}F NMR resonance assignments and experiments confirming the presence of dynamic heterogeneity within the BRAF dimer. a) ^{19}F NMR spectra of BRAF labeled on the αC-helix with BTFA in the presence of AZ628 (purple) and ATP (blue). AZ628 causes a shift from the upfield resonance to the downfield resonance, whereas ATP causes the opposite, allowing the upfield resonance to be assigned to the monomer/αC-out state and the downfield resonance to be assigned to the dimer/αC-in state. **b)** ^{19}F NMR spectra of BRAF at different BRAF concentrations. The fraction peak area of the αC-in peak versus the αC-out peak was plotted as a function of BRAF concentration (upper right) and fit to a monomer-dimer equilibrium model (blue), and was in good agreement with the model determined by FRET experiments (dotted line) with a K_D^{dimer} of 28.1 μM. **c)** ^{19}F NMR spectra of BRAF (gray) and BRAF containing dimer disrupting mutations (blue). The disappearance of the downfield resonance in the presence of the dimer disrupting mutations is consistent with the resonance assignments discussed in **a**. **d)** Linewidths extracted from deconvoluted ^{19}F NMR spectra corresponding to the individual species represented in Figure 4a. Data represent the best-fit values from the spectral deconvolution of n=8 independent experiments. **e)** ^{19}F NMR T2 relaxation profiles obtained from BRAF. Intensities at -84.42 ppm (green) and -84.29 ppm (black) corresponding to the αC-out and αC-in resonances, respectively, were plotted as a function of decay time. Relaxation profiles required a multi-exponential fit ($p<0.0001$) indicating the presence of overlapping resonances with distinct relaxation times. Data represent the mean ± s.e.m.; n=15 independent experiments. **f)** Solvent isotope effect experiments obtained with BRAF in 10% D_2O (dotted lines) and 90% D_2O (solid lines). Individual component fits from spectral deconvolution are shown and correspond to the αC-out (red), αC-in^{narrow} (yellow), and αC-in^{broad} (blue) states. Relative peak shifts indicate that the αC-in states exhibit a similar degree of solvent exposure compared to the αC-out state.

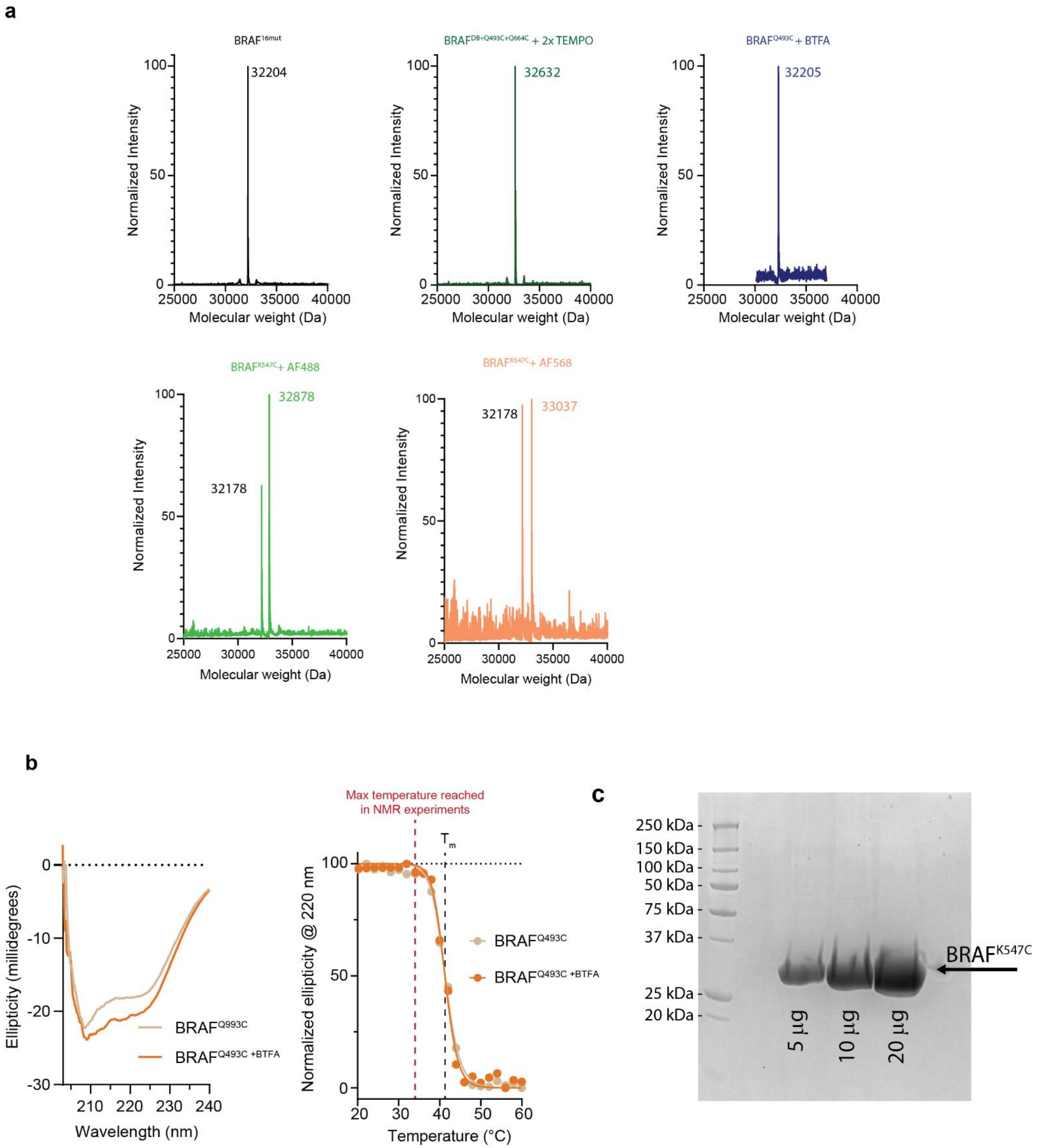

Supplementary Figure 15. Validation of BRAF labeling, stability, and purity. a) Mass spectra of BRAF^{16mut} (theoretical molecular weight: 32,204 Da), BRAF$^{DB+Q493C+Q664C}$ labeled with two 4 Maleimido-TEMPO spin probes (theoretical molecular weight: 32,136 Da), BRAF^{Q493C} labeled with 3-Bromo-1,1,1-trifluoroacetone (BTFA, theoretical molecular weight: 32,205 Da), BRAF^{K547C} (32,178 Da) labeled with AF488 (theoretical molecular weight: 32,900 Da), BRAF^{K547C} (32,178 Da) labeled with AF568 (theoretical molecular weight: 33,060 Da. **b)** Circular dichroism spectra of BRAF^{Q493C} (tan) and BRAF^{Q493C} labeled with BTFA (orange). Ellipticity at 220 nm was plotted as a function of temperature. Note that the presence of the BTFA probe does not influence the melting temperature (T_m) which is well above the maximum temperature used in variable temperature NMR experiments. **c)** SDS-PAGE gel of BRAF^{K547C} at increasing total protein amounts showing the purity of the samples used in intermolecular FRET experiments.

inhibitor	source	monoisotopic mass [M+Na]+ (Da)	measured mass [M+Na]+ (Da)	
vemurafenib	Selleck Chemicals	512.06178	512.0394	
PLX7904	Selleck Chemicals	535.1334	535.1336	αC-out
encorafenib	Selleck Chemicals	562.14101	562.1418	
Dabrafenib	Selleck Chemicals	542.0903	542.0917	
GDC0879	Selleck Chemicals	357.1322	357.1321	
SB590885	TargetMol	476.20571	476.204	αC-in type I
L779450	Selleck Chemicals	348.0898	348.0895	
Sorafenib Tosylate	Selleck Chemcials	465.0936	465.093	
TAK632	Selleck Chemcials	577.0928	577.0924	
AZ628	Selleck Chemcials	474.19	474.1904	
LY3009120	Selleck Chemcials	447.2279	447.2273	αC-in type II
Ponatinib	TargetMol	555.2091	555.2087	
ZM336372	Selleck Chemcials	412.1632	412.1631	
Belvarafenib	Selleck Chemcials	501.0671	501.0675	
MLN2480	Selleck Chemcials	527.99947	528.0014	

Supplementary Table 1. Vendor sources and mass spectrometry validation of all 15 inhibitors.
